# Chemical Fractionation, Environmental, and Human Health Risk Assessment of Potentially Toxic Elements in Soil of Industrialised Urban Areas in Serbia

**DOI:** 10.3390/ijerph18179412

**Published:** 2021-09-06

**Authors:** Dragana Pavlović, Marija Pavlović, Veljko Perović, Zorana Mataruga, Dragan Čakmak, Miroslava Mitrović, Pavle Pavlović

**Affiliations:** Department of Ecology, Institute for Biological Research “Siniša Stanković”—National Institute of Republic of Serbia, University of Belgrade, Bulevar Despota Stefana 142, Belgrade 11060, Serbia; marija.pavlovic@ibiss.bg.ac.rs (M.P.); veljko.perovic@ibiss.bg.ac.rs (V.P.); zorana.mataruga@ibiss.bg.ac.rs (Z.M.); dragan.cakmak@ibiss.bg.ac.rs (D.Č.); mmit@ibiss.bg.ac.rs (M.M.); ppavle@ibiss.bg.ac.rs (P.P.)

**Keywords:** urban soils, potentially toxic elements (PTEs), sources of PTEs, sequential extraction, mobility, health risk assessment

## Abstract

The primary focus of this research was the chemical fractionation of potentially toxic elements (PTEs) and their presence in several industrialised cities in Serbia. Furthermore, their origin, contamination levels, and environmental and human health risks were assessed. The results indicated that the examined soils were characterised by slightly higher Cu, Ni, Pb, and Zn levels than those set by European and national regulations. These elevated Cu, Pb, and Zn concentrations were caused by intensive traffic and proximity to industry, whereas the higher Ni levels were a result of the specific geological substrate of the soil in the study area. The environmental risk was found to be low and there was no enrichment/contamination of the soil with these elements, except in the case of Pb, for which moderate to significant enrichment was found. Lead also poses a potential non-carcinogenic risk to children through ingestion and requires special attention due to the fact that a significant proportion of this element was present in the tested soil samples in a potentially available form. Analysis of the health risks showed that children are more at risk than adults from contaminants and that ingestion is the riskiest exposure route. The carcinogenic risk was within the acceptable limits.

## 1. Introduction

Over the last 50 years, intensive technological and industrial development across the world has led to the development of new cities and industrial centres and the expansion of existing ones, housing more than half of today’s global population [1]. Stimulated by economic development, this trend has resulted in the generation and accumulation of various pollutants, which have a major impact on the quality of life experienced by people in the urban environment [1,2]. The main sources of pollution in such environments are combustion products originating from traffic, city heating plants, individual furnaces, industry, construction activities, and also the inadequate storage of industrial and municipal waste [3,4]. Pollutants reach the soil through wet and dry deposition, and their further fate depends on a number of physical, chemical, and biological factors [5,6]. One of the most important groups of pollutants in the urban environment are potentially toxic elements (PTEs), which are persistent and not subjected to biodegradation; this is why they accumulate in the surface layer of the soil, from where they can easily reach the food chain [1,4,6,7]. People come into contact with PTEs through ingestion, inhalation, or skin contact [1,2,8,9,10], with children particularly at risk due to their low body weight and specific habits while playing [2]. Lead can disrupt the nervous and immune systems and can cause anaemia, nephropathy, and gastrointestinal problems, while Cr exposure can lead to skin lesions, peripheral vascular diseases, and lung, skin, and liver cancer [1,2,7,11]. On the other hand, low concentrations of some PTEs (Cu, Fe, Mn, Ni, and Zn) are necessary for some basic physiological functions [6]; however, long-term exposure to high doses of Cu, Cr and Zn can adversely affect fertility, liver function, and cholesterol levels [12].

The contamination of urban soils with PTEs and their impact on human health has been the subject of numerous studies across the world [7,8]; however, more recently, research has focussed on assessing the impact of PTEs by taking into account their speciation and bioavailability [2,13,14]. In addition, every city is unique in terms of urban structure and land use patterns, which results in different PTE exposure levels for its inhabitants [7,11]. With this in mind, it is clear that examining the impact of PTEs on the environment and human health is necessary for each individual urban setting. Although PTEs are known to be harmful, even in small amounts, due to their longer residence time, toxicity, persistence, bioavailability and ubiquitous nature, scientists agree that these properties, as well as biogeogenic distribution, depend not only on their total content, but also on their chemical speciation in the soil and other factors related to the physical and chemical characteristics of the soil [3,6]. Therefore, from the point of view of environmental protection and the preservation of human health, it is far more important to determine patterns of element speciation in the soil rather than their total concentrations [15,16]. Sequential extraction methods provide a good basis for understanding the chemical properties of elements in soil and assessing potentially available forms. These methods provide information on the main binding sites and the distribution of toxic elements in different geochemical soil phases with the successive use of extraction agents of varying aggressiveness [3,17]. One widely used sequential extraction method is the BCR sequential procedure, which fractionates the PTEs in three phases: (1) exchangeable and acid-soluble (soluble in water or weakly bound to carbonates); (2) reducible (PTEs bound to iron and manganese oxides); and (3) oxidative (PTEs bound to organic matter and sulphides), with a fourth additional step—digestion with aqua regia—recommended [18,19,20], during which PTEs that are bound to the crystal structure of the mineral are released.

Research was conducted in urban parks exposed to various pollution sources in four industrial centres in Serbia (Pancevo, Smederevo, Obrenovac, and Belgrade). Parks were chosen as research sites due to the fact that the population in cities, especially children, spend most of their free time in such places. The PTEs assessed in this study (As, Cd, Co, Cr, Cu, Fe, Mn, Ni, Pb, Sr and Zn) were chosen as they are the most common pollutants of urban environments. The main objectives of this study were to: (1) quantify the content of selected PTEs in the examined urban soils; (2) determine the distribution of PTEs in different fractions using a modified BCR sequential extraction procedure, with the aim of assessing their mobility and potential bioavailability; (3) define the origin of PTEs in the soil using sequential soil extraction and statistical methods (correlation analysis (CA), principal component analysis (PCA)); (4) determine contamination levels and the potential ecological risk to the urban environment using appropriate indices (EF, Cf, Cdeg, E_r_^i^, RI); and (5) assess the impact of PTEs on the health of the local population (children and adults) by analysing the non-carcinogenic and carcinogenic health risks.

## 2. Materials and Methods

### 2.1. Study Area

**Figure 1:**
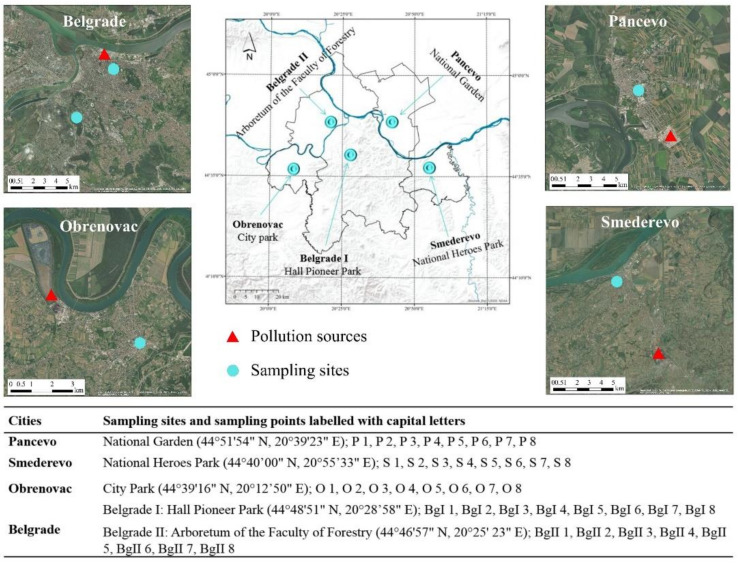
Map of the study area showing sampling sites.

The study area covers an area of about 2250 km^2^ and is located in the central part of Serbia (Figure 1). It is characterised by a temperate continental climate with warm summers and cold, dry winters. One specific aspect of the climate is the strong, cold, dry, squally south-easterly wind known as ‘kosava’, which usually blows in early spring and late autumn. This wind, together with the other dominant ones, adversely affects pollution. In terms of geology, the study area is characterised by crystalline shales, limestones, marls, Pontian and Neogene sediments, sedimentary rocks, etc., while pedagogically speaking, there are several types of soil in the study area (Chernozem; Cambisol, Eutric; Vertisol; Fluvisol; Fluvisol, Humic, and Gleysol) [21,22]. However, soils in towns and cities, particularly in urban parks, are constantly subject to various direct and indirect anthropogenic influences, meaning that, unlike natural soils, they often have a disturbed structure with altered physical, chemical and biological characteristics [23,24,25,26]. There are also differences between urban soils themselves as they can be skeletal due to filling with soil, gravel, and other waste construction materials (slag, concrete, mortar, glass, plastic, etc.), compacted due to trampling by foot or vehicular traffic, and also nitrified due to the introduction of various organic matter (paper, textile, plant parts, etc.), which becomes part of the decomposition process [27,28]. Therefore, each urban soil has its own specifics, which make it different to natural soils and hence urban soil cannot be considered as a soil type, but Urbic Technosol [24,25]. According to the classification system used in Serbia, urban soils are also classified as technogenic soils [22]. Several important industrial centres are located within this area and almost a third of Serbia’s total population lives within them.

Four industrial centres (Pancevo, Smederevo, Obrenovac, and Belgrade) were selected for this research, with the selection criteria being different sources of pollution. The dominant sources of pollution in Pancevo are an oil refinery, a nitrogen fertiliser factory and the petrochemical industry, situated in the industrial zone, and located approximately 5 km from the National Garden sampling site. The main source of pollution in Smederevo is the ironworks, located 7 km southeast of the city centre, while in Obrenovac, the dominant pollution source is the “Nikola Tesla A” thermal power plant (TENT A) with ash and slag dump sites. The sampling site in Obrenovac was in the main City Park, situated 4 km away from the source of pollution. On the other hand, traffic has been recognised as the main source of pollution in the central area of Belgrade. Emissions of pollutants from the various industrial plants in the study area result in the enrichment of soil with different PTEs; namely, the petrochemical industry emits Cd, Cr, Cu, Ni, Pb, and Zn [29,30], while iron smelters can lead to extensive contamination of surrounding soils with Cd, Cr, Ni, Pb, Zn, and other metals or metal oxides, depending on the types of scrap metals processed [31]. Thermal power plants that burn fossil fuels can emit As, B, Ba, Cd, Cr, Cu, Mo, Ni, Pb, Sr, V, and Zn [32,33,34], while emissions from traffic include Ba, Cd, Cr, Cu, Ni, Pb, Zn, and to a lesser extent As, Ag, Co, Mn, and Mo [26,35]. In each industrial centre, one representative urban park was selected where sampling was performed. The locations of the sampling sites (urban parks), including major sources of pollution are presented in Figure 1. When selecting the parks, special care was taken to ensure that they were all approximately equidistant from the main sources of pollution (4–7 km) and that the same baseline conditions applied to each of them, except for the Belgrade II sampling site. Belgrade II is the Faculty of Forestry’s *Arboretum*, which is a protected natural area and a valuable archive of local and foreign tree species in Belgrade. It is located in an area without a direct source of pollution, 10 km away from the city centre, within a zone of mixed *Quercus frainetto* and *Quercus cerris* forest. Detailed information on the sampling sites is given in the Supplementary Material to this paper.

### 2.2. Sampling and Analysis

While selecting sampling points, care was taken to ensure that sampling was even and covered the entire area of the park, i.e., that the number of samples taken (evenly distributed across the park) provided a realistic picture of the condition of the soil in that park. The soil at each sampling site was sampled from eight individual sampling points following a harmonised sampling regime. At each sampling point, five subsamples of soil were collected from a depth of 0–20 cm within an area of 1 m^2^; these were then mixed into a composite sample, thus forming 8 composite samples per park, or 40 from the entire study area. The total weight of the composite soil samples was ∼2 kg. Sampling was performed using stainless steel equipment and tools, taking care to exclude larger stones, gravel and other foreign objects from the sampling. The surface layer of soil (0–20 cm) was chosen for analysis because toxic metal deposition in soil in urban areas mostly occurs in top soil [20]. The collected samples were packed in labelled plastic bags and taken to the laboratory, where they were dried to a constant weight, crushed and sieved through a 2 mm nylon mesh sieve. Samples prepared in this way were used for further analysis.

Soil texture was determined by the combined pipette method with 0.4 N solution of sodium pyrophosphate (Na_4_P_2_O_7_), with fractionation performed according to Atterberg, and texture classes determined using the International Soil Texture Triangle [36] and presented as percentages of sand, silt, and clay (%). As regards the chemical properties of the soil, this included the electrometric determination of the soil pH in H_2_O (1:2.5 soil-distilled water ratio) as well as in 1 M KCl (1:2.5 soil–KCl ratio) using a WTW (Germany) inoLab^®^ 7110 pH meter with a glass electrode, calibrated at pH 3, pH 7, and pH 12 with buffer solutions (WTW, Germany). Levels of organic carbon (OC) and nitrogen (N) were determined using the method developed by Nelson and Sommers [37] involving the dry burning of samples at 1150 °C using a CNS Vario EL III analyser (Germany). The Scheibler calcimeter method was used to determine CaCO_3_ content, while the OC content was obtained as the difference between total and inorganic carbon.

Chemical fractionation of PTEs in soil was conducted using the optimised three-stage extraction procedure recommended by BCR [19,38,39] in closed 50 cm^3^ polyethylene tubes. The three-phase BCR extraction protocol and the determination of pseudo total metal contents are described below:

Step 1: 40 cm^3^ of acetic acid (0.11 mol dm^−3^) was added to 1.000 ± 1 mg of soil in a 50 cm^3^ polyethylene tube and shaken for 16 h (overnight) at room temperature in an end-over-end mechanical shaker operating at 30 rpm. The extract was separated from solid residue by centrifugation at 3.000× *g* for 10 min. The supernatant was decanted and diluted to 50 mL with 1 M nitric acid and stored in a polyethylene bottle at 4 °C until metal analysis. The residue was washed by adding 10 cm^3^ of deionised water, shaking for 10 min and finally centrifuging for 10 min at 3.000× *g*. The supernatant was decanted and discarded, acting cautiously so as not to discard any solid residues.

Step 2: 40 cm^3^ of freshly prepared hydroxylamine hydrochloride (0.5 mol dm^−3^, pH 1.5) was added to the residue from Step 1 and extraction was performed as described above.

Step 3: 10 cm^3^ of hydrogen peroxide (8.8 mol dm^−3^) was added carefully in small aliquots to the residue from Step 2. The vessel was loosely covered with the watch glass and digested by heating to 85 °C in a water bath for 1 h; then the volume was reduced to a few ml by further heating of the uncovered vessel. A second 10 mL of aliquot of hydrogen peroxide was added and the digestion procedure was repeated. Moreover, 40 cm^3^ of ammonium acetate (1 mol dm^−3^, adjusted to pH 2 with nitric acid) was added to the cool moist residue, which was extracted as described above. The solid residue was retained for aqua regia digestion.

Step 4: pseudo total metal content or aqua regia digestion involves the digestion of soils with a mixture of mineral acids (3:1, *v*/*v*, hydrochloric acid to nitric acid) in a water bath. A total of 8 cm^3^ of aqua regia was carefully added to the residue from Step 3 and digested by heating to 85 °C in a water bath for 1 h. A second 8 cm^3^ of aliquot of aqua regia was added and the digestion procedure was repeated. After aqua regia digestion and cooling, the extract was filtered through a Munktell filter no. 389, and diluted with 1 M nitric acid to a volume of 50 cm^3^ and stored in a polyethylene bottle at 4 °C until metal analysis. Digestion of the residual material is not specified in the BCR protocol. Blank extractions, i.e., without soil, were carried out following the complete procedure for each set of analyses and using the same reagents. Concentrations of the extracted elements were measured using optical emission spectrometry for simultaneous multi-element analysis (ICP—OES, Spectro Genesis). Pseudo-total element concentrations in the soil samples were calculated as the sum of the PTE concentrations in all four fractions. Concentrations of the chemical elements were expressed in mg kg^−1^ of soil. Analytical procedures were validated using standard reference material (BCR 701, Institute for Reference Materials and Measurements (IRMM), Geel, Belgium) for three-step sequential extraction; this was treated in the same way as the samples for the purposes of the quality control of laboratory protocols. The accuracy of the obtained results was in the range of 100 ± 15% (Table S1 provided in Supplementary Material), demonstrating a high level of agreement between the measured and certified values. Each sample was analysed in triplicate and standard deviations of the triplicate measurements were less than 10%. Detection limits (mg kg^−1^) for the elements were as follows: Co—0.001, Cr—0.011, Cu—0.007, Fe—0.011, Mn—0.001, Ni—0.029, Pb—0.001, Sr—0.001 and Zn—0.004.

### 2.3. Environmental and Ecological Risk Assessment

The degree of soil pollution as well as the possible impact of anthropogenic activities on the content of‘ PTEs in soil was assessed using the enrichment factor (EF), contamination factor (Cf) and degree of contamination (Cdeg), while the degree of environmental risk caused by PTEs was assessed using potential ecological risk (E_r_^i^) and the potential ecological risk index (RI). The impact of PTEs on the health of children and adults was assessed by calculating the non-carcinogenic and carcinogenic health risk for children and adults.

Enrichment factor (EF)

The enrichment factor (EF) is a useful geochemical tool for assessing the degree of the anthropogenic impact on the level of soil pollution by PTEs [40]. Equation (1) is used to calculate EF:

(1) $$\mathrm{EF}=\left(C_{i}/C_{r}\right)/\left(B_{i}/B_{r}\right)$$

where Ci is the measured pseudo-total concentration of the tested element in the soil sample, Cr—the pseudo-total concentration of the reference element in the soil sample, Bi—the background concentration of the tested element and Br—the background concentration of the reference element. Fe was chosen as the reference element because of its immobility, crustal abundance and high stability in the earth’s crust [41,42]. Background values of PTEs in the studied soils were proposed by Mrvić et al. [43], Mrvić et al. [44] and Knežević [45]. The arithmetic median method was used to calculate background concentrations (the median absolute deviation (MAD) from the data’s median) [44,46] for each sampling site (Table S2, provided in Supplementary Material).

Contamination factor (Cf)

The contamination factor is the ratio of the pseudo-total concentrations of the tested PTE (C_i_) in the study area and its pre-industrial, i.e., background, values (C_b_), and is used to monitor the enrichment of PTEs in soils over a period of time [47]. Cf is calculated using Equation (2):

(2) $$\mathrm{Cf}=C_{i}/C_{b}$$

Degree of contamination (Cdeg)

The degree of contamination (Cdeg) represents the sum of the pollution factors [47] and is calculated using Equation (3):

(3) $$C_{\deg}=\sum_{i=1}^{n}\mathrm{Cf}$$

Potential ecological risk ($E_{r}^{i}$)

The potential ecological risk ($E_{r}^{i}$) of a particular PTE is the product of the contamination factor (Cf) and the toxic response factor ($T_{r}^{i}$) of a particular PTE and is calculated using Equation (4):

(4) $$E_{r}^{i}=T_{r}^{i}\times \mathrm{Cf}$$

$T_{r}^{i}$ reflects the level of toxicity and biological sensitivity to contamination by a particular element. $T_{r}^{i}$ values are as follows: Cu—5, Cr—2, Pb—5, Ni—5, Zn—1 [47].

Potential Ecological Risk Index (RI)

The Potential Ecological Risk Index (RI) represents the sum of potential ecological risks [47] and is calculated using Equation (5):

(5) $$\mathrm{RI}=\sum_{i=1}^{n}E_{r}^{i}$$

The recommended classifications for all the factors and indices used are given in Table S3 (provided in Supplementary Material).

### 2.4. Health Risk Assessment

An assessment of the impact of PTEs on human health (children and adults) was conducted in accordance with the recommendations of the U.S. Environmental Protection Agency [48]. When assessing the non-carcinogenic and carcinogenic health risk, three basic routes of PTE intake were considered—ingestion, inhalation, and dermal contact [42]. Health risks were assessed for each of the examined sites separately, with calculations taking into account various coefficients related to residents [49]. A quantitative assessment of the non-carcinogenic risk from PTEs in soil was performed using hazard quotients (HQs) for each examined PTE [9,10], while the carcinogenic risk (CRs) was only quantified for those elements with defined slope factors.

Non-carcinogenic risk

(6) $$\mathrm{HQing}=\left[\left(C\times \mathrm{IRS}\times \mathrm{RBA}\times \mathrm{EF}\times \mathrm{ED}\right)/\left(\mathrm{BW}\times \mathrm{AT}\times \mathrm{RfDo}\right)\right]\times 10^{-6}$$

(7) $$\mathrm{HQder}=\left[\left(C\times \mathrm{SA}\times \mathrm{AF}\times \mathrm{ABSd}\times \mathrm{EF}\times \mathrm{ED}\right)/\left(\mathrm{BW}\times \mathrm{AT}\times \mathrm{RfDo}\times \mathrm{GIABS}\right)\right]\times 10^{-6}$$

(8) $$\mathrm{HQinh}=\left[\left(C\times \mathrm{EF}\times \mathrm{ED}\right)/\left(\mathrm{AT}\times \mathrm{RfC}\times \mathrm{PEF}\right)\right]$$

The hazard index (HI) is used to estimate the total non-carcinogenic risk for the three exposure pathways and is calculated using the following equation:

(9) HI=HQing+HQder+HQinh

However, as HQs and HI refer to the risks of one specific PTE, their cumulative (total) values were calculated for a more complete picture; this represents the total impact of all the tested elements for each exposure route (CHQs) and the impact of all the elements through all the exposure routes together (CHI) [10]. CHQs and CHI are calculated using the following equations:

(10) $$\mathrm{CHQing}=\sum_{i=1}^{n}\mathrm{HQing}$$

(11) $$\mathrm{CHQder}=\sum_{i=1}^{n}\mathrm{HQder}$$

(12) $$\mathrm{CHQinh}=\sum_{i=1}^{n}\mathrm{HQinh}$$

(13) CHI=∑HI

Carcinogenic risk

In line with the recommendations listed in [49], the carcinogenic risk for residents is calculated using the following equations:

(14) $$\mathrm{CRing}=\left(C*\mathrm{IFS}*\mathrm{RBA}*\mathrm{CSFo}\right)/\mathrm{AT})\times 10^{-6}$$

(15) $$\mathrm{IFS}=\left(\mathrm{EF}\times \mathrm{EDa}\times \mathrm{IRSa}/\mathrm{BWa}\right)+\left(\mathrm{EF}\times \mathrm{EDc}\times \mathrm{IRSc}/\mathrm{BWc}\right)$$

(16) $$\mathrm{CRder}=\left[\left(C\times \mathrm{DFS}\times \mathrm{ABSd}\times \mathrm{CSFo}\right)/\left(\mathrm{AT}\times \mathrm{GIABS}\right)\right]\times 10^{-6}$$

(17) $$\mathrm{DFS}=\left(\mathrm{EF}\times \mathrm{EDa}\times \mathrm{SAa}\times \mathrm{AFa}/\mathrm{BWa}\right)+\left(\mathrm{EF}\times \mathrm{EDc}\times \mathrm{SAc}\times \mathrm{AFc}/\mathrm{BWc}\right)$$

(18) CRinh=C×EF×ED×IUR×1000/AT×PEF

The total carcinogenic risk (TCR) is the sum of the carcinogenic risks for each of the three exposure routes and is calculated using the following equation:

(19) TCR=CRing+CRder+CRinh

The carcinogenic risk due to the combined effects of all the tested elements for the three exposure routes is calculated following the same principle as the non-carcinogenic risk, using the following equations:

(20) $$\mathrm{CCRing}=\sum_{i=1}^{n}\mathrm{CRing}$$

(21) $$\mathrm{CCRder}=\sum_{i=1}^{n}\mathrm{HQder}$$

(22) $$\mathrm{CCRinh}=\sum_{i=1}^{n}\mathrm{CRinh}$$

(23) CTCR=∑TCR

If HQ and HI values are less than 1, no adverse effects on health are expected [42,50], while acceptable values for CR are considered to be in the range of 10^−4^ to 10^−6^ [12,42,50]. A description and reference values for all the parameters in the above equations are given in Tables S4 and S5, while the calculations of HQ, HI, and CR were performed using USEPA’s RSL calculator [51].

### 2.5. Statistical Analysis

One-way analysis of variance (ANOVA) was performed in order to distinguish the differences in PTE content in the soil samples between sampling sites in the study area (subsequent tests of normality using the Shapiro–Wilk W test and Levene’s test of homogeneity of variances showed non-significant values for all the reported ANOVA breakdowns). Pearson’s correlation coefficient (p) and Principal Component Analysis (PCA) were used to determine the relationships between the elements in the soil and to determine their potential origin using the SPSS Version 21 software package (IBM, Chicago, IL, USA) [52]. In order to obtain a clearer picture of the relationships between the examined variables, the basic components were rotated using the Varimax rotation method with Kaiser normalisation [42].

## 3. Results and Discussion

### 3.1. Selected Physical and Chemical Soil Properties

The selected physical and chemical characteristics of the examined soils, i.e., the soil texture, the soil pH in an aqueous solution (pH_H2O_), the potential acidity (pH_KCl_), and the amount of organic carbon (OC) and nitrogen (N), are presented in Table 1.

Soil texture is a very important factor since a whole range of physical (structure, porosity, water–air, heat) and chemical (adsorption and buffering capacity) properties depend on it [4]. The studied soils can be classified mainly as clay loam and sandy clay loam, according to the USDA classification [36]. The lowest total sand content (38.70%) and the highest clay content (38.43%) was measured at the Belgrade II sampling site, while the highest total sand content (53.01%) and the lowest clay content (25.43%) was measured in Smederevo. The silt fraction varied from 21.57% in Smederevo to 27.70% in Obrenovac. Moghtaderi et al. [53] found urban soils in Shiraz (Southwest Iran) to have a more marked heterogeneity in terms of texture, with the sand content ranging from 21.9–72.6%, silt from 21.57–27.70%, and clay from 7.6–33.5%. It is well known that a fine-grained soil fraction exhibits a higher tendency for PTE adsorption than coarse-grained soils since it contains soil particles with large surface areas such as clay minerals, iron, and manganese oxy-hydroxides, and humic acids [54]. Based on a specifically built triangle for soil classification according to texture, the dominant soil type at the Pancevo, Obrenovac, and Belgrade II sampling sites was clay loam, while sandy clay loam was identified in Smederevo and at Belgrade I. Dragović et al. [31] also classified the soils around the ironworks in Smederevo as silty clay loam and silty loam. The pH_H2O_ of the soil solution fell within a narrow range—between 7.92 at the Belgrade II sampling site and 8.62 at Belgrade I, which classifies them as moderately alkaline [36]. The pH_KCl_ solution was relatively uniformly distributed and ranged from 6.85 in Pancevo to 7.10 in Belgrade, classifying these soils as practically neutral [36]. Soil pH controls the geochemical behaviour of those elements, which are present in the solid and soluble phases of the soil and directly affects the processes of sorption/desorption, uptake and chemical speciation of the elements present in the soil [55,56]. Soil alkalinity is a common occurrence in urban soils and is most often the result of using alkalising products such as calcium carbonate and calcium–magnesium carbonate in gravel, cement, and concrete, but is also attributed to the deposition of alkaline atmospheric particles and carbon of anthropogenic origin, from coal combustion [28,57,58]. Rarely, urban soils can also be acid soils since the nature of the geological parent material has a very important role [26]. High alkalinity is a negative feature of a substrate and often causes a deficiency of essential micronutrients [28], but it can also have a positive effect in terms of the immobilisation of potentially toxic, labile forms of elements that form permanent complexes with organic matter in the soil [4,58].

Organic carbon and nitrogen in soil play a key role in many biogeochemical and pedogenetic processes, as they impact its fertility and have a direct effect on soil-plant interactions [59,60]. The origin of OC in soil is twofold: it occurs naturally through the decomposition of organic matter and anthropogenically from various human activities [4,61]. The anthropogenic inflow of OC cannot compensate for or replace the natural inflow from the mineralisation of organic matter because in this way the chemical structure of the soil changes [61]. In this study, the highest OC content was measured at the Belgrade I sampling site (4.05%), which is located near very busy roads in one of the most polluted parts of Belgrade, and can be linked to its anthropogenic inflow. The OC content in the parks in other cities and municipalities was significantly lower and fell within a narrow range—from 2.05% in Obrenovac to 2.80% in Pancevo. Moghtaderi et al. [53] found high variability in OC levels in urban soils in Shiraz (Southwest Iran), where its content ranged from 0.27–5.5%. Nitrogen in soil originates from the mineralisation of organic matter and its amount depends directly on the inflow of organic matter, which is why soils with a higher percentage of OC have a higher content of nitrogen compounds [60]. This is confirmed by the results of this study, where the highest percentage of N was measured at the sampling site with the highest content of OC—Belgrade I (0.31%).

### 3.2. Pseudo-Total Content of Selected PTEs in Examined Urban Soils

Basic statistics and differences between the content of PTEs in the examined soils are shown in Table 2 and Table S6. In order to compare the content of PTEs in the examined soils with their content in the upper continental crust, and with the threshold values of PTEs, as set by European and national legislation, the following were used: the composition of the continental crust [62], Finnish legislation on contaminated soils [63], and regulations on allowable concentrations of hazardous and harmful substances in the soil [64]. After examining the options presented by the various approaches and the thresholds they apply, the authors chose the standards set by Finnish legislation on contaminated soils. The Finnish standard values are a good approximation of the mean values of different national systems in Europe [65].

As concentrations of As and Cd were below the detection level in all of the analysed samples, they were not discussed any further. On the basis of the obtained results, it can be concluded that PTE content at all the investigated sites was below levels in the upper continental crust [62], threshold values for European soils [63], and maximum allowable concentrations of PTEs as set by national legislation [64,66], except for Ni and Pb (Table 2). The highest levels of Co, Fe, and Mn were measured in soil samples from Obrenovac, Cr and Ni in Smederevo, and Cu, Pb, Sr, and Zn in Belgrade. Cobalt content at Belgrade II (10.75 mg kg^−1^) and Obrenovac (11.75 mg kg^−1^) exceeded standard levels of 9 mg kg^−1^ set by national legislation [66]. The content of Co in Obrenovac differs significantly in comparison to all the other sampling sites, with the exception of Belgrade II. However, the lowest level, measured at Belgrade I, is statistically different only from those concentrations measured in Obrenovac and at Belgrade II (Table 2). The average Cr, Fe, Sr, and Zn levels in the selected soil samples were within the proposed limits according to European and national legislation [62,63,64]. Somewhat elevated Cu concentrations were measured in samples from Smederevo and Belgrade I, which was statistically confirmed (Table 2), but these were still at a level that does not pose a danger to the environment, given the alkalinity of the soil and slightly higher OC content at these sites [67]. The average Mn content in the examined soils was higher than its average content in the surface soils of Europe (382 mg kg^−1^, ref. [68]). The content of Ni in soils varies considerably and depends primarily on its content in the parent rock [69]. In the examined soils, its average content varied from 44.60 mg kg^−1^ at the Belgrade I sampling site to 104.05 mg kg^−1^ in Smederevo. Statistical analysis showed that Ni content in Smederevo differed significantly in comparison to the other sampling sites. Such results for Ni content in soil are expected, given that numerous studies have confirmed that higher concentrations of Ni are characteristic for the soils of Serbia [14,69,70,71]. During research spanning several decades, these authors have proven that the main source of Ni in the soil is actually the parent rock, i.e., the minerals it is composed of [69]. Čakmak et al. [14] believe that the elevated Ni levels are linked to areas with intensive soil erosion, which occurs due to heavy rainfall, as a result of which it is washed into the basins of larger rivers (the Kolubara and the Morava) and then transported by them [70,72].

The results of one-way ANOVA showed that there was a statistically significant difference (*p* < 0.001, *p* < 0.01, *p* < 0.05) in the content of all PTEs at the examined sites, apart from Pb (ns). The lack of significance for Pb occurs due to the high heterogeneity of its content in the examined soils.

A comparison of PTE content in urban soils in some Serbian cities and European urban soils is shown in Table 3.

When comparing results of PTEs content obtained in this research with results from our previous research [4,73], levels of Mn were found to be similar, but Cr, Cu, Ni, Pb, and Zn concentrations were higher. On the other hand, Cr, Cu, Pb, and Zn content in soils in Novi Sad [74], Lisbon [79], and Thessaloniki [73] was similar, but Ni was somewhat lower than the levels measured in this research (Table 2). Similarities in terms of PTE content were also found in soils from Šabac [75] and Kragujevac [76], where concentrations of most of the examined elements were similar to those determined in this research, with the exception of the higher content of Cr in Kragujevac and Mn in Šabac. Soils in Warsaw [82] and Prague [81] are characterised by a lower content of Co, Cr, Cu, Mn, Ni, and Pb than in the examined soils in cities in Serbia, while soils in Turin [83] and Zagreb [77] have a higher content. In addition, soils in Zagreb [77], Turin [83] and Vigo City [26], are characterised by a higher Zn content than soils in other European cities, and soils in Sopron [78] by a higher Cu and Pb content. In terms of Sr, its content in the examined soils was lower than in soils in Zagreb [77], but higher than in Vigo City [26]. Based on these observations, it can be concluded that urban soils exhibit exceptional heterogeneity in terms of the presence and content of PTEs. This is to be expected, given the fact that urban soils have undergone significant changes and contain varying amounts of different additional materials, such as those used in the construction of buildings or roads, and also the fact that the surface layer (0–20 cm), which is most often the focus of research, is continuously exposed to intensive pollutants from a range of sources.

### 3.3. Partitioning of PTEs in Examined Urban Soils

**Figure 2:**
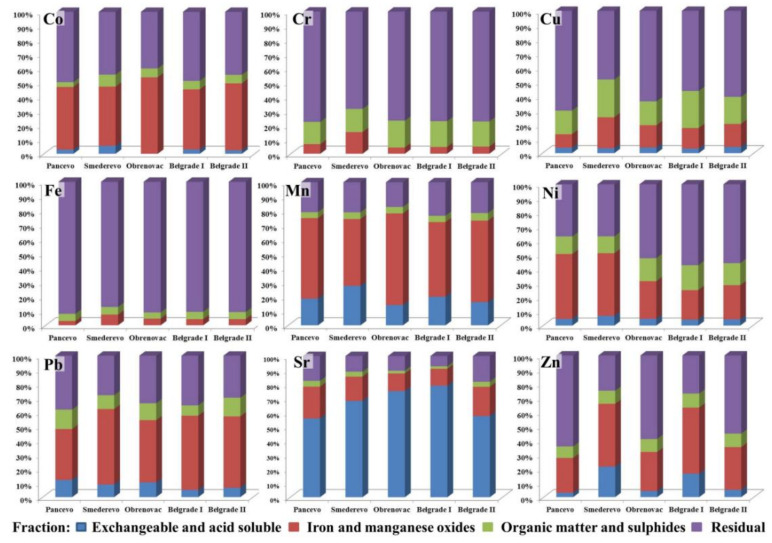
Fractionation profile of PTEs in the examined soils.

The fractionation profiles of the selected PTEs in the examined soils are shown in Figure 2 and Figures S1–S3 (provided in Supplementary Material).

Generally, Co content in European soils does not reach levels that could pose a risk to the environment [84]. Despite the fact that Co in soils originates mainly from parent materials, it can also come from anthropogenic sources, above all from mining and smelting activity, fertiliser use and sewage sludge spreading [85], which can apply to the samples from Obrenovac. However, the absence of Co extracted in the acid-soluble/exchangeable fraction in the samples from Obrenovac, as well as a significant amount bound to Fe and Mn oxides, points unequivocally to its geological origin, especially if we bear in mind the fact that Co in soil is closely correlated with elements of geological origin [86], as confirmed by both correlation and PCA in this study: Co-Fe (0.828 **) and Co-Mn (0.810 **). Furthermore, in the alkaline conditions found at all the sampling sites, Co would be expected to immobilise. However, the fractionation profile of Co reveals a similar share in its distribution between the reducible (35–60%) and residual fractions (35–58%), while only a small proportion was bound to organic matter and sulphides (1–12%). A reduction in soil Eh and pH can lead to the solubilisation of precipitated or adsorbed Co [87].

Chromium can be present in soil in different oxidation states, the most important from the environmental point of view being Cr (VI) and Cr (III), as the most stable and dominant forms of Cr in nature, and its speciation depends on redox potential, soil pH, the presence of potential electron donors and Cr adsorption by colloids [13,56,88]. It is believed that most soil Cr occurs as Cr (III), and is incorporated within the mineral structures. Chromium (III) and its compounds are considered to be very stable and are mainly found bound to organic matter in soils; at pH 5.5 it is almost completely precipitated [88,89]. Sequential analysis confirmed that Cr was stable and firmly bound to the crystal structure of the mineral, with most extracted in the residual fraction (68.63–77.70%), while the remainder was bound to organic matter and sulphides (15.71–18.67%) and Fe and Mn oxides (4.33–14.84%). The slightly higher percentage share in the reducible (14.84%) and oxidisable (16.48%) fractions in Smederevo probably represents the combined influence of the geological substrate and the way park surfaces are formed. These results are in line with the results of Wang et al. [15], who found that in geologic serpentine soils Cr is mainly found in the oxidisable and residual fractions (over 90%), while this share is somewhat lower in anthropogenically contaminated soils. As Cr in soil is mainly present in the insoluble Cr (III) form, it is very immobile, which, together with the alkaline reaction of soil, means the risk to humans is low [15]. This is also important from the environmental point of view because Cr is a potentially toxic element, yet in this form, it does not pose a serious ecological risk [6,56].

Sequential extraction results for Cu indicated a possible anthropogenic impact in Smederevo and at Belgrade I, where approximately 50% of Cu was extracted in the first three fractions, while the rest was bound to primary and secondary soil minerals. At the other sampling sites, Cu was mainly bound to soil minerals (60.68–70.12%), while the rest was bound to organic matter and sulphides (16.31–18.79%) and Fe and Mn oxides (9.47–16.08%), with only a small proportion (3.89–4.43%) extracted in the exchangeable and acid-soluble fraction. It is known that Cu shows high binding affinity for various soil components, particularly organic matter; in addition, the stability and speciation of Cu depend greatly on pH and its mobility decreases with increasing pH [67]. Thus, it can be concluded that Cu in the examined soils is stable and poorly mobile, while the samples from Smederevo and Belgrade I exhibited slightly higher mobility and bioavailability, with a certain amount of Cu coming from anthropogenic sources (traffic and ironworks). Prolonged exposure to high Cu levels damages the liver, kidneys and brain, which can lead to hepatitis, coronary heart disease, and psychological and neurological symptoms [87]. Copper, together with Pb and Zn, is a pollutant that is associated with the wear and tear of tyres and brake pads and engine oil decomposition [35], which is confirmed by the significant positive correlation of Cu-Pb (0.796 **), Cu-Zn (0.841 **), and Pb-Zn (0.750 **).

Given the fact that Fe is one of the most common elements in the lithosphere, its standard, threshold and target values are not defined by European and national legislation [64,66,90]. Iron content in the examined soils ranged from 27.66 g kg^−1^ at Belgrade I to 34.64 g kg^−1^ in Smederevo. The dynamics of Fe in soil are closely linked to the cycling of O, S, and C, as well as to the Eh–pH system of the external environment and the oxidative phase of the Fe compounds that are present [91]. In this sense, Fe is characterised by fast and easy changes in its oxidation state and its chemical behaviour is similar to Co and Ni [87]. Lindsay and Norwell [92] state that the solubility of inorganic Fe depends on soil pH, with it being lowest in the pH 6.5–8.0 range, which was also the case with the examined soils. In the existing conditions, the solubility and mobility of inorganic Fe is reduced to a minimum, as confirmed by results of sequential extraction, which revealed the strong binding of Fe to silicate and oxide soil minerals (87–92%), indicating the fact that Fe is of geological origin. This is supported by the results of sequential analysis, where the share of Fe in the reducible (3.01–7.54%) and oxidisable (4.25–5.16%) fractions was similar, while it was insignificant in the acid-soluble fraction (0.01–0.02%).

**Figure 3:**
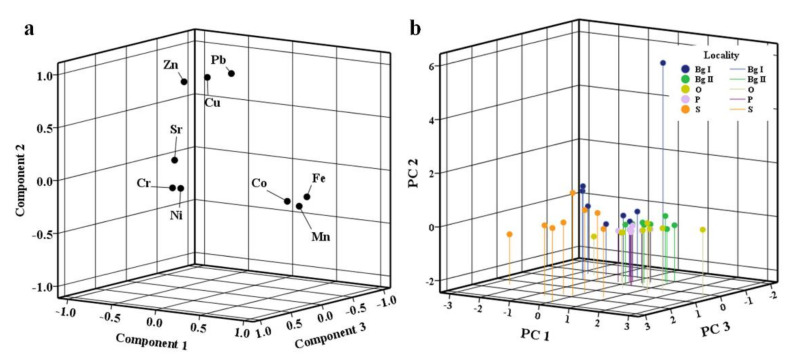
PCA for selected PTEs in the examined soils: (**a**) loading plot, (**b**) score plot.

Manganese in urban soils mainly comes from lithogenic sources, but can also be a product of various anthropogenic activities, such as the smelting of metal ores, emissions during steel and iron alloy production, the combustion of fossil fuels, and, to a lesser extent, emissions during the combustion of fuel additives [93,94]. Specifically, in the exhaust gases from cars, the organic Mn compound methyl-cyclopentadienyl-manganese-tricarbonyl (MMT) can be found, which is one of the main alternatives to lead additives in gasoline [35,94]. In addition, ash from coal combustion in thermal power plants may contain significant amounts of this element [95], which is probably the cause of its elevated levels in Obrenovac, where the dominant source of pollution is a thermal power plant. Manganese is an essential element, but chronic exposure to Mn can lead to serious disorders, which is why it is considered extremely toxic to human health, and it can have a negative impact on the ecosystem due to its accumulation in the food chain [95]. Despite this, the positive impact of Mn can be seen in its oxides due to their potential to sorb heavy metals in the soil and to act as oxidants, thus positively affecting soil vitality [96]. Sequential extraction showed that the largest share of Mn was extracted in the reducible fraction (46.42–63.13%), followed by the exchangeable, acid-soluble (14.89–27.86%), and residual (17.50–23.60%) fractions, while the least was bound to organic matter and sulphides (4.16–5.34%). Such a fractionation profile is expected since Mn deposited in soil tends to be bound to oxides, carbonates and silicates [35], i.e., in the soil it is mainly in the form of Mn (II), which is its water-soluble and exchangeable form, and in the form of insoluble Mn oxides (Mn (IV)) [96,97]. The primary targets of hydroxylamine hydrochloride are specifically Mn oxides and hydroxides [18]. Keeping in mind the results of the correlation and PCA (Figure 3), the alkalinity of the examined soils, and the fractionation profile, it can be concluded that Mn in the tested soils is stable and does not pose a danger to the environment. However, any change in pH and redox potential can increase its availability, which would adversely affect the environment.

Elevated Ni concentrations require a more cautious approach given its potential to promote cancer mortality in humans, with this risk depending on its mobility and bioavailability in soil, as only bioavailable fractions can be absorbed by humans [15]. Sequential analysis of Ni showed that, at the sites in Obrenovac and Belgrade, it is predominantly incorporated in the crystal structure of the mineral (52.74–57.52%), and as such, does not pose a health risk. Of the non-residual fractions, especially in Pancevo and Smederevo, Ni was primarily held in the reducible fraction (the Fe-Mn oxide fraction) (43.02–45.62%). Antić-Mladenović et al. [71] point out that Fe-Mn (hydro) oxides act as scavengers for soil Ni and that a fractionation profile such as this one represents a characteristic pattern for the lithogenic origin of metals in soils [98]. The high proportion of Ni in these two fractions can also be linked to Ni-bearing clay minerals, which can be formed during ultramafic rock weathering and can be further transported by rivers [14,99]. The smallest part of Ni was extracted within the exchangeable fraction (4.39–6.92%), which implies that under current native conditions, Ni is stable. Fe-Mn oxides are thermodynamically stable in alkaline conditions [71], which were encountered in our study; however, any change in redox potential, pH, salinity, microbial activity or hypoxic conditions can lead to the release of soluble Ni [100]. For this reason, most authors do not exclude the possibility of anthropogenic contamination of soil with Ni, along with its natural enrichment. In our study, the geological origin of Ni was also confirmed by correlation analysis whereby strong positive correlations with Co (0.349 *) and Cr (0.954 **) were found, elements for which other researchers also determined significant positive correlations and a predominantly geological origin [14].

Numerous previous studies on Pb content in urban soils have indicated variations in Pb content depending on the position of the sampling site, with the highest concentrations determined in intensive traffic zones [35,101]. Lead is a common pollutant of urban soils and its origin is most often associated with anthropogenic activities, i.e., emissions from traffic and industry, with the anthropogenic impact on soils in cities being more marked due to the higher population density, traffic intensity and proximity to industrial plants. The extremely high content of Pb at Belgrade I is probably the result of past emissions from motor vehicles. Specifically, although the practice of deliberately adding Pb to fuel was halted worldwide long ago [35], leaded gasoline was produced in Serbia until 2010 [20,102] and traces of it can still be found in fuel [35]. In addition, the low mobility and high adsorption capacity of Pb for different soil components hinders a reduction in its concentrations in the surface layer of soil yet further [35,101]. In Smederevo, Pb content (99.11 mg kg^−1^) was at the very top end of maximum permitted concentrations prescribed by national legislation (100 mg kg^−1^, ref. [64]), pointing to the obvious impact of emissions from the ironworks, but also from traffic. The results of sequential extraction confirmed the anthropogenic origin of Pb, given that in the soils of Smederevo and Belgrade Pb was predominantly bound to Fe and Mn oxides and hydroxides, i.e., that more than 75% was extracted in the first three fractions, and a significantly smaller part was bound to soil minerals. A fractionation profile such as this indicates that Pb can be easily mobilised if external conditions change, which is a risk to the environment, especially if we bear in mind the fact that ingestion of soil and dust is one of the important pathways of Pb exposure, especially in children [84,101]. Chronic effects that occur due to the long-term intake of lead into the body include neurological and gastrointestinal problems, anaemia, damage to the kidneys, endocrine, and immune systems, and disorders in the psychophysical development of children [1,7]. At the other sampling sites, Pb was approximately equally distributed between the residual and reducible fractions, with a significant share being extracted at these sites in the first three phases of extraction (>60%), indicating the presence of anthropogenic sources at these sites as well, although their impact is somewhat less pronounced (Figure S3, provided in Supplementary Material). The results of the correlation analysis support this assertion regarding the origin of Pb, i.e., there was a significant positive correlation between Cu, Pb and Zn, which are elements that originate from traffic, with Pb-Cu (0.796 **) and Pb-Zn (0.750 **).

Strontium is an alkaline earth element, which can be found in the environment in a wide range of concentrations as a result of natural mineral degradation or the constant impact of anthropogenic pollution [103,104]. Due to its marked reactivity, it does not occur in nature in its elemental form, but most often as a mineral, carbonate (strontianite), or sulphate (Celestine). Its natural content in soil depends almost exclusively on the climatic conditions and the composition of the parent rock, while the average content ranges from 175–458 mg kg^−1^ [105]. Strontium is found in small quantities in association with other alkaline earth metals that; have similar properties to Ca, which is why it usually competes with them. From the environmental point of view, this can be a significant problem because this similarity with Ca conditions its fast uptake; it is easy transport to aboveground plant parts, and its involvement in the cycling of matter [89]. Once it enters the body, Sr easily gets into the bloodstream, mimicking and behaving very much like Ca. It can easily be incorporated into bones, especially in children, where it can create hard bone mineral and lead to problems with bone growth or the weakening of bones. The removal of Sr from the body is a very long and, slow process [106]. Strontium is known for its mobility and is derived mainly from leachable minerals such as feldspars, apatite, and micas [68]; hence, why it causes concern in terms of ecosystems [104]. Existing data from literature indicates that the majority of Sr is retained in the surface soil layer [20,107] and is, therefore, mainly associated with the exchangeable fraction in soil, which was confirmed by our study (49.32–76.75%). The second largest fraction is related to Fe and Mn oxides (12.77–25.87%), which indicates the high mobility of Sr in the examined soils and its potential toxicity. The share of Sr in the residual fraction was between 8.48% and 21.32%, while it was least associated with organic matter and sulphides (1.99–4.69%). These results concur with those of Stefanović et al. [17], who determined that Sr is one of the most easily mobilised elements. Data obtained from correlation and PCA showed that the origin of Sr was different from that of all the other examined elements. Strontium negatively correlated with elements of geological origin such as Co (−0.319 *), Fe (−0.493 **), and Mn (−0.539 **), which indicates its anthropogenic origin (traffic, individual furnaces, the use of various artificial substrates in parks).

The main source of Zn in soil is the parent substrate on which the soil is formed; however, in urban soils, in addition to the parent substrate, Zn can originate from various anthropogenic sources. Copper, Pb, and Zn in urban soils usually have a common origin and are often referred to as ‘urban metals’ [108], as confirmed by research based on GIS-based and multivariate statistical analyses showing that urban soils are characterised by elevated concentrations of Cu, Pb, and Zn with the dominant pollution sources being traffic, the use of paints and various industrial emissions [109,110]. The influence of anthropogenic sources on Zn content, i.e., the impact of traffic and emissions of suspended particles from the ironworks, was determined in this study, with the highest content of this element measured in Smederevo (114.82 mg kg^−1^) and at Belgrade I (135.33 mg kg^−1^). Sequential extraction also confirmed the anthropogenic impact at these sites, with Zn in the samples from Smederevo and Belgrade I being mainly exchangeable and associated with carbonates (14.0–20.31%), and Mn and Fe oxides primary and secondary soil minerals (56.43–64.75%), followed by its binding to Mn and Fe oxides (24.30–29.16%), while only a small portion was extracted in the oxidisable (8.07–9.52%) and acid-soluble/exchangeable fractions (2.88–4.88%). The significant portion of Zn in the reducible fraction indicates the importance of Fe and Mn oxides for the accumulation of this element, which is confirmed by the similar fractionation profile of these two elements. Based on all the above, it can be concluded that the anthropogenic impact is most pronounced in Smederevo and at Belgrade I, and also that Cu, Pb, and Zn are of common origin.

### 3.4. Environmental and Ecological Risk Assessment

Values of selected pollution indices are shown in Table 4 and Tables S7–S10 (provided in Supplementary Material).

Based on the average enrichment factor (EF) values obtained (Table 4), it was determined that there was no enrichment with the examined PTEs in the soils in the study area. The average EF values for all the PTEs at all the sampling sites were at the level of minimal enrichment, except for Pb, for which moderate enrichment was determined in Pancevo (2.84) and significant enrichment at Belgrade I (7.73) (Table S3, provided in Supplementary Material). However, although the results indicated that there was no notable soil enrichment with the tested PTEs, EF values > 1.5 for Cu and Zn at Belgrade I and Pb at all the sampling sites point to their possible anthropogenic origin [111], while EF values > 1.5 for Co and Ni arise as a result of the impact of the geological substrate on their concentrations in soil in this part of Serbia [112]. It was also noticed that there was no regularity in terms of PTE soil enrichment increasing or decreasing, which points to the heterogeneity of the examined soils.

The average values of the contamination factor (Cf) for Co, Cu, Cr, Mn, Ni, Sr, and Zn were less than 1 at all the sampling sites, except for Cu in Smederevo (1.13), Fe in Smederevo (1.23), at Belgrade I (1.12) and Belgrade II (1.34), Mn at Belgrade II (1.12), Ni in Pancevo (1.24), and Sr in Obrenovac (1.09) and at Belgrade I (1.57), Table 4 and Table S3 (provided in Supplementary Material). For Co and Pb, the average Cf value was higher than 1 at all the sampling sites, with extremely high Cf values for Pb at Belgrade II (8.84), which indicates that the soil at this site is impacted anthropogenically (Table S3, provided in Supplementary Material). Higher Cf values for Pb compared to the other elements can be put down to high contamination levels in soil due to both natural and anthropogenic sources [9]. Therefore, it can be concluded that the examined soils are not polluted, but that there is a potential risk in the case of Pb, especially if its high share in the first three fractions of sequential extraction (the bioavailable fractions) is taken into account. The contamination factor is a useful indicator of the impact of individual PTEs on soil pollution; however, to consider the overall impact of PTEs on soil pollution, it is also necessary to calculate the degree of contamination (Cdeg). The average Cdeg values obtained at all the sampling sites except for Belgrade I were < 8, which indicates a low level of contamination. At Belgrade I, Cdeg values were at the level of a moderate degree of contamination (15.21), (Table 4 and Table S3, provided in Supplementary Material), which shows unequivocally that the anthropogenic impact is felt most keenly in Belgrade and is most likely the result of the elevated Pb content.

The single ecological risk factor (Ei) revealed that only Pb was at the level of ‘medium risk’ at Belgrade I (44.19); however, this did not notably affect the overall ecological risk, which was at a low level at all sampling sites (Table 4 and Table S3, provided in Supplementary Material).

### 3.5. Health Risk Assessment

Non-carcinogenic risk values through ingestion (HQ_ing_) for children and adults decreased in the following order: Fe > Pb ≥ Co > Mn > Cr > Ni > Cu > Zn > Sr and were less than 1 for all elements, indicating that there was no significant non-carcinogenic risk. The exception is HQ_ing_ values for Pb at Belgrade I, where this element presents a significantly higher non-carcinogenic risk for children than the other tested PTEs (Table 5). This result can be explained by the elevated Pb concentrations at this site, as well as by its high intoxication and respective RfD value [40]. Similar high HQ_ing_ values for Pb were also obtained by Jia et al. [7]. This result deserves special attention due to the fact that the amount of potentially available Pb at this site is greater than 75%. However, despite the fact that the HQ_ing_ values for each element were classified as ‘acceptable’, their cumulative values (CHQ_ing_) for children were significantly greater than 1 at all sites, which indicates unequivocally that the investigated PTEs in the studied soils pose a significant non-carcinogenic risk through the ingestion exposure route. The highest CHQ_ing_ values for both children and adults were determined in soil samples from Belgrade I (Table 5 and Table 6). The values for the non-carcinogenic risk through dermal contact (HQ_der_) decreased in the order of Mn ≥ Cr > Ni > Fe ≥ Pb ≥ Co > Cu > Zn > Sr for both children and adults, while values for the non-carcinogenic risk through inhalation (HQ_inh_) followed the order: Mn > Co > Ni > Cr > Pb > Cu > Zn for both children and adults. Unlike CHQ_ing_, values for both individual HQ_der_ and HQ_inh_ and for cumulative CHQ_der_ and CHQ_inh_ were less than 1, indicating that PTEs in soil pose no significant non-carcinogenic risk when it comes to the dermal and inhalation exposure routes. When considering the results for the non-carcinogenic risk for the three exposure pathways, it was observed that ingestion has the higher potential risk for children and adults, which is in accordance with previous research by Jia et al. [7], Wu et al. [113] and Jadoon et al. [9]. Health index (HI) values for children and adults decreased in the order: Fe > Pb > Co > Mn > Cr > Ni > Cu > Zn > Sr. However, although the individual HI values for children and adults were less than 1, their total, cumulative values (CHI) for children were above the maximum acceptable values at all sampling sites, with the non-carcinogenic risk through ingestion being the main contributing factor (HQ_ing_) at over 90%, which is in accordance with previous findings [113,114]. The highest CHI values were at Belgrade I. Based on these results, it is clear that residents are exposed to the greatest risk through ingestion and that of all the examined PTEs, Pb potentially represents the greatest risk. Adding to this risk is the fact that a significant portion of Pb in the examined soils is in a potentially available form (Figure 2). Comparing the HI values for children and adults, it was noted that those for children were almost 10 times higher, which points to the greater sensitivity of children to the detrimental health effects of PTEs in soils compared to adults. Numerous previous studies have also highlighted the increased sensitivity of children to the effects of PTEs [7,10,42,113,114], with their specific hygiene-related habits during play further increasing this impact [2,9].

The carcinogenic risk (CR) values for Co, Cr, Ni, and Pb and the cumulative carcinogenic risk (CCR) through ingestion, dermal contact, and inhalation pathways were in the ‘acceptable’ range (10^−4^—10^−6^, ref. [50]), indicating that there is no danger of cancer, Table 7. The CCR values for all three exposure pathways decreased in the following order: CCR_ingestion_ > CCR_dermal_ > CCR_inhalation_, which is in accordance with the results of Varol et al. [10]. CCR_ing_ accounts for the largest share of the total cumulative cancer risk (CTCR), being as much as 10 times higher than CCR_der_ and almost 100 times higher than CCR_inh_. In terms of the results of the carcinogenic risk by sampling site, it was noticed that although the values were ‘acceptable’, the residents of Smederevo are at potentially the greatest risk of getting the some type of illness. It was further observed that Cr accounts for the largest share of CTCR by ingestion, dermal contact, and inhalation, which is in accordance with the findings of Varol et al. [10]. However, sequential analysis showed that Cr in the examined soils is stable and firmly bound to the crystal structure of the mineral, which reduces its potentially negative effects.

### 3.6. Determining the Origin and Relationship between PTEs

Based on the correlation analysis results, the examined elements were divided into three groups. The first group consisted of Co, Fe, and Mn, which exhibited a significant positive correlation with each other (Table 8), but also a significant negative correlation with Sr and Zn. The second group included Cu, Pb, and Zn, which were significantly positively correlated with each other, with only Zn being significantly negatively correlated with Co, Fe, and Mn. The third group consisted of positively correlated Cr and Ni, with Ni also positively correlated with Co. Strontium displayed behaviour that was opposite to the other examined elements and it could not be put into any of these groups, i.e., it exhibited a significant negative correlation with Co, Fe, and Mn. A similar relationship between the elements in urban soils has been found in five European cities with a significant positive correlation between Cu, Pb, and Zn, and a positive correlation between Cr and Ni [108]. These authors revealed the anthropogenic origin of Pb, Zn, and Cu and the natural origin of Cr and Ni. Other studies have yielded similar results [115,116].

PCA results were in accordance with correlation analysis results, i.e., three components were singled out, which together explained 85.09% of the variability (Table 9, Figure 3a). The first component (41.92%) represented the influence of the geological substrate and exhibited a significant positive correlation with Co, Fe, and Mn, and a significant negative one with Sr. The second component (26.09%) represented the anthropogenic impact, i.e., the impact of traffic, given that the wear and tear of brake pads, tyres, and various parts of the car leads to the release of Cu, Pb, and Zn into the environment [2,35], and it is precisely these elements that Component II had the strongest influence on. The third component (17.08%) represented the combined influence of the geological substrate and the way park surfaces are formed, with Cr and Ni correlating with it. Elevated Cr and Ni levels is one of the specific features of alluvial soils [2,14], which were probably used for the filling and formation of park areas due to their wide availability in the investigated localities. Based on the results of correlation and PCA, it was established that Sr did not belong to any of the determined groups of elements and that this element is influenced by an additional factor that is not statistically significant (eigenvalues < 1), but which indicates that Sr is of different origin to the other examined elements, especially when compared to those elements of geological origin (Co, Fe, and Mn), with which it correlates negatively (Table 8).

Spatially speaking, samples from Obrenovac, Pancevo, and Belgrade II stand out as being under the influence of the first component (PC1) in particular, while in Obrenovac the influence of PC3 is also present (Figure 3b). Samples from Belgrade are strongly impacted by PC2, while samples from Smederevo are influenced by PC3 and PC2. Hence, it can be concluded that the anthropogenic influence is dominant at Belgrade I and, to some extent, in Smederevo, while in Pancevo, Obrenovac and at Belgrade II the dominant impact is geological. The way parks are formed impacts the samples from Smederevo and partly from Obrenovac, too. Park soils are formed for that specific purpose by using substrates of different origins and composition; in addition, they are under the constant influence of anthropogenic factors, which makes them very heterogeneous and explains the differences that existed between samples collected from the same site.

## 4. Conclusions

The results of the research show elevated concentrations of Cu, Pb, Ni, and Zn in relation to national and European legislation. Excessive Ni is a result of the specific geological substrate on which the soil was formed and cannot be linked to anthropogenic contamination, while excess Cu, Pb, and Zn is a result of anthropogenic activities. In terms of mobility, the following order was determined by chemical fractionation: Sr > Mn > Pb > Zn > Ni > Cu > Co > Cr > Fe. Most Sr was extracted in the acid-soluble exchangeable fraction, and most Mn and Pb in the reducible one. The distribution of Co, Ni, and Zn varied between the reducible and residual fractions depending on the site, while the residual fraction was dominant for Fe, Cr, and, to a slightly lesser extent, for Cu.

The environmental and ecological risk assessments showed that there was no enrichment or contamination of the soil with the examined PTEs, except in the case of Pb, for which moderate to significant enrichment was found, which was especially pronounced at the Belgrade I sampling site. This result did not significantly affect the overall ecological risk, which was low at all sites.

Hazard quotient (HQ) values for each of the tested PTEs were at the acceptable level, except for HQ_ing_ for Pb at Belgrade I. However, the cumulative hazard quotient values for the ingestion exposure route (CHQ_ing_) for children were significantly higher than 1 at all the sites, indicating unequivocally that the joint impact of the investigated PTEs in the studied soils poses a significant non-carcinogenic risk through the ingestion pathway. Hazard index (HI) values for children and adults were also less than 1, while at all sites their total values (THI) for children were above the maximum acceptable values. Based on these results, it is clear that children are most at risk through ingestion, and that of all the PTEs examined, Pb poses potentially the greatest risk. Carcinogenic risk (CRs and TCR) values were in the ‘acceptable’ range, indicating that there is no risk of cancer. It was also established that the largest contribution to total TTCR by ingestion, dermal contact, and inhalation is from Cr; however, since Cr in the examined soils is stable and firmly bound to the crystal structure of the mineral, its potentially negative effects are minimised.

Furthermore, it was determined that Co, Fe, and Mn are of natural geological/lithogenic origin, Cu, Pb, and Zn are anthropogenic, mainly from traffic and the ironworks, and the origin of Cr and Ni is related to the geological substrate and the way parks are formed. Results of PCA also showed that Sr does not belong to any of these categories of elements and that it is influenced by an additional factor that is not statistically significant, but which points to it being of different origin than the other examined PTEs, probably anthropogenic.

This research showed that geological substrate and the way the parks are formed had the greatest influence on the content of PTEs in the examined soils. While the impact of traffic and industry is evident, this is not to an extent that would cause a serious ecological problem. For children, there is a significant non-carcinogenic risk of exposure to Pb through ingestion, with its elevated content being the result of ‘historical contamination’ of the soil, and this should decrease over time.

## Figures and Tables

**Table 1:** Selected physical and chemical parameters of sampled urban soils.

Sampling Site	Depth (cm)		Granulometric Composition %						
		Soil Texture	Total Sand	Silt	Clay	pH		O C	N
			2.0–0.02 mm	0.02–0.002 mm	<0.002 mm	H_2_O	KCl	%	%
Pancevo	0–20	Clay Loam	44.78	26.76	27.47	8.44	6.85	2.79	0.28
Smederevo	0–20	Sandy Clay Loam	53.01	21.57	25.43	8.54	6.98	2.61	0.22
Obrenovac	0–20	Clay Loam	41.07	27.70	31.24	8.56	7.06	2.05	0.15
Belgrade I	0–20	Sandy Clay Loam	47.82	22.81	29.38	8.63	7.10	4.06	0.31
Belgrade II	0–20	Clay Loam	38.70	22.87	38.43	7.92	6.93	2.38	0.25

**Table 2:** Pseudo-total concentrations and basic descriptive statistics of PTEs in the examined soils and a comparison with European and national regulations and average values in the upper continental crust (average values of PTEs above the MAC are denoted in bold; average values above the threshold value for European soils are underlined).

Sampling Site	mg kg^−1^	Co	Cr	Cu	Fe	Mn	Ni	Pb	Sr	Zn
Pancevo	average	8.76 ^bc^	40.97 ^b^	31.12 ^b^	32,843.09 ^ab^	570.46 ^ab^	**61.35** ^bc^	46.56 ^a^	42.46 ^b^	45.56 ^b^
	min	8.12	36.17	28.47	31,093.92	499.21	51.10	40.24	24.08	38.17
	max	9.25	48.37	34.48	34,410.49	611.98	76.92	55.86	100.59	56.62
	stdev	0.40	4.26	2.05	1044.44	37.26	8.47	4.90	25.01	6.74
Smederevo	average	9.46 ^bc^	77.85 ^a^	48.54 ^a^	29,435.41 ^ab^	508.51 ^bc^	**104.05 ** ^a^	99.11 ^a^	70.06 ^b^	114.82 ^a^
	min	6.72	49.46	30.58	18,117.88	342.22	57.41	57.27	57.21	86.47
	max	12.09	98.82	84.07	40,037.83	597.23	134.34	200.18	89.46	168.71
	stdev	1.54	14.81	15.63	6039.52	77.63	24.19	49.56	11.54	25.40
Obrenovac	average	11.75 ^a^	45.73 ^b^	33.54 ^b^	34,641.16 ^a^	640.29 ^a^	**75.69 ** ^b^	53.26 ^a^	84.58 ^ab^	48.30 ^b^
	min	8.13	28.20	23.81	25,939.71	453.31	51.32	40.44	31.45	34.82
	max	14.69	56.72	42.95	41,141.78	912.85	92.20	69.21	155.60	70.94
	stdev	1.99	9.66	5.42	4560.19	135.17	14.55	8.84	43.23	11.53
Belgrade I	average	8.34 ^c^	27.48 ^c^	49.84 ^a^	27,668.93 ^b^	452.01 ^c^	44.60 ^c^	**327.03 ** ^a^	122.24 ^a^	135.33 ^a^
	min	6.48	23.28	33.84	23,315.14	391.74	38.37	52.69	62.20	53.70
	max	9.25	35.89	107.36	29,738.63	546.83	61.07	1750.38	190.49	302.36
	stdev	0.92	4.03	24.71	2349.24	51.26	7.31	579.97	49.93	82.70
Belgrade II	average	10.32 ^ab^	34.17 ^bc^	34.47 ^b^	33,272.35 ^a^	548.17 ^abc^	**50.97 ** ^c^	61.42 ^a^	43.94 ^b^	48.55 ^b^
	min	8.77	22.78	29.42	29,409.41	441.45	34.55	46.72	28.45	37.80
	max	11.35	39.84	44.31	36,066.42	627.79	67.75	94.13	92.49	68.23
	stdev	0.72	5.50	4.66	2253.07	67.35	10.36	15.88	21.63	9.52
*p* Value		***	***	*	**	**	***	ns	***	***
Threshold value for European soils [63]		20	100	100	/	/	50	60	/	200
MAC [64]		/	100	100	/	/	50	100	/	300
Upper continental crust [62]		17.3	92	28	39,200	774	47	17	320	67

Values are average with standard deviation (stdev), *n* = 8; individual values of pseudo-total element concentrations have been provided in Table S6 (provided in Supplementary Material). ANOVA, mean (SD), levels of significance: *** *p* < 0.001, ** *p* < 0.01, * *p* < 0.05, ns—not significant. Different letters in the same column indicate significant differences between sampling sites.

**Table 3:** Comparison of PTE content in urban soils in some Serbian cities and European urban soils.

City	Country	Co	Cr	Cu	Mn	Ni	Pb	Sr	Zn	References
Pancevo	Serbia		87.3	20.9	676.4				54.7	[4]
Smederevo	Serbia		126.0	64.1	595.6				151.3	[4]
Obrenovac	Serbia		93.3	20.9	691.9				65.7	[4]
Belgrade	Serbia		72.1	19.5	523.9				72.4	[4]
Belgrade	Serbia		32.3	34.9		37.46	53.3		61.6	[73]
Novi Sad	Serbia	7.3	28.0	38.8	368.6	28.7	82.3		100.3	[74]
Šabac	Serbia	11.0	59.8	37.6	70.1	47.6	82.0			[75]
Kragujevac	Serbia		744.2	30.9	434.9		91.5		31.7	[76]
Zagreb	Croatia	21.0	72.7	63.8	959.0	39.6	85.2	134.0	178.0	[77]
Sopron	Hungary	20.6		118.4		25.7	124.5		132.9	[78]
Lisbon	Portugal		55.6			34.6	84.1			[79]
Thessaloniki	Greece		32.2	39.7		40.65	42.7		48.3	[73]
Athens	Greece	16.0	163.0	48.0	587.0	111.0	77.0		122.0	[80]
Salzburg	Austria		20.9	38.3		23.05	87.70		66.2	[73]
Prague	Czech Republic		36.4	19.1		21.5	23.5		106	[81]
Warsaw	Poland	4.0		35.0		29.5	29.5		96.5	[82]
Vigo City	Spain		68.5	66.1	531.55	32.01	96.3	58.8	149.0	[26]
Turin	Italy	23.0	270	90.0		240.0	220.0		216.0	[83]

**Table 4:** Environmental and ecological risk assessment of PTEs in the examined soils using enrichment factor (EF), contamination factor (Cf), degree of contamination (Cdeg), ecological risk index (Ei), and potential ecological risk (RI).

	EF								
Sampling Site/Elements	Co	Cr	Cu	Mn	Ni	Pb	Sr	Zn	
Pancevo	1.996	1.321	1.262	1.268	1.907	2.842	0.835	0.726	
Smederevo	1.113	0.945	0.687	0.598	0.839	1.532	0.434	0.850	
Obrenovac	1.132	0.684	1.427	0.877	0.685	1.816	1.012	0.778	
Belgrade I	1.107	0.407	1.493	0.827	0.560	7.727	0.501	1.801	
Belgrade II	1.142	0.419	0.860	0.832	0.530	1.251	0.149	0.533	
	Cf								Cdeg
Sampling site/elements	Co		Cu	Mn	Ni	Pb	Sr	Zn	
Pancevo	1.302	7.810	0.862	0.827	1.244	1.854	0.546	0.290	7.810
Smederevo	1.357	8.776	1.128	0.725	1.010	1.853	0.901	0.410	8.776
Obrenovac	1.342	7.321	0.579	0.741	0.578	1.516	1.087	0.219	7.321
Belgrade I	1.237	15.207	0.451	0.924	0.619	8.839	1.571	0.171	15.207
Belgrade II	1.531	7.976	0.560	1.120	0.708	1.660	0.565	0.145	7.976
	Ei								RI
Sampling site/elements		Cr	Cu		Ni	Pb		Zn	
Pancevo		18.664	1.159		6.220	9.270		0.290	18.664
Smederevo		17.788	0.808		5.051	9.263		0.410	17.788
Obrenovac		13.946	2.099		2.889	7.582		0.219	13.946
Belgrade I		49.752	1.390		3.097	44.193		0.171	49.752
Belgrade II		14.824	1.720		3.539	8.300		0.145	14.824

**Table 5:** Non-carcinogenic (HQ, CHQ, HI, and CHI) risk for children through ingestion, inhalation, and dermal contact exposure pathways.

HQing	Co	Cr ^a^	Cu	Fe	Mn	Ni	Pb	Sr	Zn	CHQing
Pancevo	3.73 × 10^−1^	1.75 × 10^−1^	9.95 × 10^−3^	6.00 × 10^−1^	3.04 × 10^−1^	3.92 × 10^−2^	4.25 × 10^−1^	9.05 × 10^−4^	1.94 × 10^−3^	1.93
Smederevo	4.03 × 10^−1^	3.32 × 10^−1^	1.55 × 10^−2^	5.38 × 10^−1^	2.71 × 10^−1^	6.65 × 10^−2^	9.05 × 10^−1^	1.49 × 10^−3^	4.89 × 10^−3^	2.54
Obrenovac	5.01 × 10^−1^	1.95 × 10^−1^	1.07 × 10^−2^	6.33 × 10^−1^	3.41 × 10^−1^	4.84 × 10^−2^	4.86 × 10^−1^	1.80 × 10^−3^	2.06 × 10^−3^	2.22
Belgrade I	3.55 × 10^−1^	1.17 × 10^−1^	1.59 × 10^−2^	5.05 × 10^−1^	2.41 × 10^−1^	2.85 × 10^−2^	2.99	2.60 × 10^−3^	5.77 × 10^−3^	4.26
Belgrade II	4.40 × 10^−1^	1.46 × 10^−1^	1.10 × 10^−2^	6.08 × 10^−1^	2.92 × 10^−1^	3.26 × 10^−2^	5.61 × 10^−1^	9.36 × 10^−4^	2.07 × 10^−3^	2.09
HQder	Co	Cr ^a^	Cu	Fe	Mn	Ni	Pb	Sr	Zn	CHQder
Pancevo	8.86 × 10^−4^	1.66 × 10^−2^	2.36 × 10^−5^	1.42 × 10^−3^	1.80 × 10^−2^	2.33 × 10^−3^	1.01 × 10^−3^	2.15 × 10^−6^	4.61 × 10^−6^	4.03 × 10^−2^
Smederevo	9.57 × 10^−4^	3.15 × 10^−2^	3.68 × 10^−5^	1.28 × 10^−3^	1.61 × 10^−2^	3.95 × 10^−3^	2.15 × 10^−3^	3.54 × 10^−6^	1.16 × 10^−5^	5.59 × 10^−2^
Obrenovac	1.19 × 10^−3^	1.85 × 10^−2^	2.54 × 10^−5^	1.50 × 10^−3^	2.02 × 10^−2^	2.87 × 10^−3^	1.15 × 10^−3^	4.28 × 10^−6^	4.88 × 10^−6^	4.55 × 10^−2^
Belgrade I	8.43 × 10^−4^	1.11 × 10^−2^	3.78 × 10^−5^	1.20 × 10^−3^	1.43 × 10^−2^	1.69 × 10^−3^	7.09 × 10^−3^	6.18 × 10^−6^	1.37 × 10^−5^	3.63 × 10^−2^
Belgrade II	1.04 × 10^−3^	1.38 × 10^−2^	2.61 × 10^−5^	1.44 × 10^−3^	1.73 × 10^−2^	1.93 × 10^−3^	1.33 × 10^−3^	2.22 × 10^−6^	4.91 × 10^−6^	3.69 × 10^−2^
HQinh	Co	Cr ^a^	Cu	Fe	Mn	Ni	Pb	Sr	Zn	CHQinh
Pancevo	1.03 × 10^−3^	2.89 × 10^−4^	9.14 × 10^−6^		8.04 × 10^−3^	4.81 × 10^−4^	2.19 × 10^−5^		9.18 × 10^−7^	9.88 × 10^−3^
Smederevo	1.11 × 10^−3^	5.49 × 10^−4^	1.43 × 10^−5^		7.17 × 10^−3^	8.15 × 10^−4^	4.66 × 10^−5^		2.31 × 10^−6^	9.71 × 10^−3^
Obrenovac	1.38 × 10^−3^	3.22 × 10^−4^	9.85 × 10^−6^		9.03 × 10^−3^	5.93 × 10^−4^	2.50 × 10^−5^		9.73 × 10^−7^	1.14 × 10^−2^
Belgrade I	9.80 × 10^−4^	1.94 × 10^−4^	1.46 × 10^−5^		6.37 × 10^−3^	3.49 × 10^−4^	1.54 × 10^−4^		2.73 × 10^−6^	8.07 × 10^−3^
Belgrade II	1.21 × 10^−3^	2.41 × 10^−4^	1.01 × 10^−5^		7.73 × 10^−3^	3.99 × 10^−4^	2.89 × 10^−5^		9.78 × 10^−7^	9.62 × 10^−3^
HI	Co	Cr ^a^	Cu	Fe	Mn	Ni	Pb	Sr	Zn	CHI
Pancevo	3.75 × 10^−1^	1.91 × 10^−1^	9.98 × 10^−3^	6.01 × 10^−1^	3.30 × 10^−1^	4.20 × 10^−2^	4.26 × 10^−1^	9.07 × 10^−4^	1.95 × 10^−3^	1.98
Smederevo	4.05 × 10^−1^	3.64 × 10^−1^	1.56 × 10^−2^	5.39 × 10^−1^	2.94 × 10^−1^	7.13 × 10^−2^	9.07 × 10^−1^	1.50 × 10^−3^	4.91 × 10^−3^	2.60
Obrenovac	5.03 × 10^−1^	2.14 × 10^−1^	1.08 × 10^−2^	6.34 × 10^−1^	3.70 × 10^−1^	5.19 × 10^−2^	4.88 × 10^−1^	1.81 × 10^−3^	2.06 × 10^−3^	2.28
Belgrade I	3.57 × 10^−1^	1.28 × 10^−1^	1.60 × 10^−2^	5.07 × 10^−1^	2.61 × 10^−1^	3.06 × 10^−2^	2.99	2.61 × 10^−3^	5.78 × 10^−3^	4.30
Belgrade II	4.42 × 10^−1^	1.60 × 10^−1^	1.11 × 10^−2^	6.09 × 10^−1^	3.17 × 10^−1^	3.49 × 10^−2^	5.62 × 10^−1^	9.38 × 10^−4^	2.08 × 10^−3^	2.14

HQ—hazard quotient; CHQ—cumulative HQ; HI—hazard index; CHI—cumulative HI. Values > 1 are in bold. ^a^ Cr(VI).

**Table 6:** Non-carcinogenic (HQ, CHQ, HI, and CHI) risk for adults through ingestion, inhalation, and dermal contact exposure pathways.

HQing	Co	Cr ^a^	Cu	Fe	Mn	Ni	Pb	Sr	Zn	CHQing
Pancevo	3.50 × 10^−2^	1.64 × 10^−2^	9.32 × 10^−2^	5.62 × 10^−2^	2.85 × 10^−2^	3.68 × 10^−2^	3.99 × 10^−2^	8.48 × 10^−2^	1.82 × 10^−2^	1.81 × 10^−2^
Smederevo	3.78 × 10^−2^	3.11 × 10^−2^	1.45 × 10^−2^	5.04 × 10^−2^	2.54 × 10^−2^	6.24 × 10^−2^	8.49 × 10^−2^	1.40 × 10^−2^	4.59 × 10^−2^	2.38 × 10^−2^
Obrenovac	4.70 × 10^−2^	1.83 × 10^−2^	1.01 × 10^−2^	5.93 × 10^−2^	3.20 × 10^−2^	4.54 × 10^−2^	4.56 × 10^−2^	1.69 × 10^−2^	1.93 × 10^−2^	2.08 × 10^−2^
Belgrade I	3.33 × 10^−2^	1.10 × 10^−2^	1.49 × 10^−2^	4.74 × 10^−2^	2.26 × 10^−2^	2.67 × 10^−2^	2.80 × 10^−2^	2.44 × 10^−2^	5.41 × 10^−2^	3.99 × 10^−2^
Belgrade II	4.12 × 10^−2^	1.37 × 10^−2^	1.03 × 10^−2^	5.70 × 10^−2^	2.74 × 10^−2^	3.05 × 10^−2^	5.26 × 10^−2^	8.78 × 10^−2^	1.94 × 10^−2^	1.96 × 10^−2^
HQder	Co	Cr ^a^	Cu	Fe	Mn	Ni	Pb	Sr	Zn	CHQder
Pancevo	1.48 × 10^−4^	2.76 × 10^−3^	3.94× 10^−6^	2.37 × 10^−4^	3.01 × 10^−3^	3.88 × 10^−4^	1.68 × 10^−4^	3.58 × 10^−7^	7.69 × 10^−7^	6.72 × 10^−3^
Smederevo	1.60 × 10^−4^	5.25 × 10^−3^	6.14× 10^−6^	2.13 × 10^−4^	2.68 × 10^−3^	6.58 × 10^−4^	3.58 × 10^−4^	5.91 × 10^−7^	1.94 × 10^−6^	9.33 × 10^−3^
Obrenovac	1.98 × 10^−4^	3.09 × 10^−3^	4.24× 10^−6^	2.50 × 10^−4^	3.38 × 10^−3^	4.79 × 10^−4^	1.93 × 10^−4^	7.13 × 10^−7^	8.15 × 10^−7^	7.59 × 10^−3^
Belgrade I	1.41 × 10^−4^	1.85 × 10^−3^	6.31× 10^−6^	2.00 × 10^−4^	2.38 × 10^−3^	2.82 × 10^−4^	1.18 × 10^−4^	1.03 × 10^−6^	2.28 × 10^−6^	6.05 × 10^−3^
Belgrade II	1.74 × 10^−4^	2.31 × 10^−3^	4.36× 10^−6^	2.41 × 10^−4^	2.89 × 10^−3^	3.22 × 10^−4^	2.22 × 10^−4^	3.71 × 10^−7^	8.19 × 10^−7^	6.16 × 10^−3^
HQinh	Co	Cr ^a^	Cu	Fe	Mn	Ni	Pb	Sr	Zn	CHQinh
Pancevo	1.03 × 10^−3^	2.89 × 10^−4^	9.14× 10^−6^		8.04 × 10^−3^	4.81 × 10^−4^	2.19 × 10^−5^		9.18 × 10^−7^	9.88 × 10^−3^
Smederevo	1.11 × 10^−3^	5.49 × 10^−4^	1.43× 10^−5^		7.17 × 10^−3^	8.15 × 10^−4^	4.66 × 10-^5^		2.31 × 10^−6^	9.71 × 10^−3^
Obrenovac	1.38 × 10^−3^	3.22 × 10^−4^	9.85× 10^−6^		9.03 × 10^−3^	5.93 × 10^−4^	2.50 × 10-^5^		9.73 × 10^−7^	1.14 × 10^−2^
Belgrade I	9.80 × 10^−4^	1.94 × 10^−4^	1.46× 10^−5^		6.37 × 10^−3^	3.49 × 10^−4^	1.54 × 10-^4^		2.73 × 10^−6^	8.07 × 10^−3^
Belgrade II	1.21 × 10^−3^	2.41 × 10^−4^	1.01× 10^−5^		7.73 × 10^−3^	3.99 × 10^−4^	2.89 × 10-^5^		9.78 × 10^−7^	9.62 × 10^−3^
HI	Co	Cr ^a^	Cu	Fe	Mn	Ni	Pb	Sr	Zn	CHI
Pancevo	3.62 × 10^−2^	1.94 × 10^−2^	9.46× 10^−4^	5.65 × 10^−2^	3.95 × 10^−2^	4.55 × 10^−3^	4.01 × 10^−2^	8.52 × 10^−5^	1.84 × 10^−4^	1.97 × 10^−1^
Smederevo	3.91 × 10^−2^	3.69 × 10^−2^	1.47× 10^−3^	5.06 × 10^−2^	3.52 × 10^−2^	7.71 × 10^−3^	8.53 × 10^−2^	1.41 × 10^−4^	4.63 × 10^−4^	2.57 × 10^−1^
Obrenovac	4.85 × 10^−2^	2.17 × 10^−2^	1.02× 10^−3^	5.96 × 10^−2^	4.44 × 10^−2^	5.61 × 10^−3^	4.58 × 10^−2^	1.70 × 10^−4^	1.95 × 10^−4^	2.27 × 10^−1^
Belgrade I	3.44 × 10^−2^	1.30 × 10^−2^	1.51× 10^−3^	4.76 × 10^−2^	3.13 × 10^−2^	3.30 × 10^−3^	2.81 × 10^−1^	2.45 × 10^−4^	5.46 × 10^−4^	4.13 × 10^−1^
Belgrade II	4.26 × 10^−2^	1.62 × 10^−2^	1.05× 10^−3^	5.72 × 10^−2^	3.80 × 10^−2^	3.78 × 10^−3^	5.28 × 10^−2^	8.81 × 10^−5^	1.96 × 10^−4^	2.12 × 10^−1^

HQ—hazard quotient; CHQ—cumulative HQ; HI—hazard index; CHI—cumulative HI. ^a^ Cr(VI).

**Table 7:** Carcinogenic (CR, CCR, TCR, and CTCR) risk for residents through ingestion, inhalation, and dermal contact exposure pathways.

CRing	Co	Cr ^a^	Ni	Pb	CCRing
Pancevo		2.71 × 10^−5^		5.24 × 10^−7^	2.77 × 10^−5^
Smederevo		5.16 × 10^−5^		1.12 × 10^−6^	5.27 × 10^−5^
Obrenovac		3.03 × 10^−5^		6.00 × 10^−7^	3.09 × 10^−5^
Belgrade I		1.82 × 10^−5^		3.68 × 10^−6^	2.19 × 10^−5^
Belgrade II		2.26 × 10^−5^		6.92 × 10^−7^	2.33 × 10^−5^
CRder	Co	Cr ^a^	Ni	Pb	CCRder
Pancevo		3.05 × 10^−6^		8.68 × 10^−8^	3.14 × 10^−6^
Smederevo		5.80 × 10^−6^		1.85 × 10^−7^	5.99 × 10^−6^
Obrenovac		3.41 × 10^−6^		9.93 × 10^−8^	3.51 × 10^−6^
Belgrade I		2.05 × 10^−6^		6.09 × 10^−7^	2.66 × 10^−6^
Belgrade II		2.55 × 10^−6^		1.14 × 10^−7^	2.66 × 10^−6^
CRinh	Co	Cr ^a^	Ni	Pb	CCRinh
Pancevo	1.90× 10^−8^	8.30 × 10^−7^	4.44 × 10^−9^	1.35 × 10^−10^	8.54 × 10-07
Smederevo	2.05× 10^−8^	1.58 × 10^−6^	7.53 × 10^−9^	2.87 × 10^−10^	1.61 × 10^−6^
Obrenovac	2.55× 10^−8^	9.27 × 10^−7^	5.48 × 10^−9^	1.54 × 10^−10^	9.58 × 10^−7^
Belgrade I	1.81× 10^−8^	5.57 × 10^−7^	3.23 × 10^−9^	9.47 × 10^−10^	5.79 × 10^−7^
Belgrade II	2.24× 10^−8^	6.92 × 10^−7^	3.69 × 10^−9^	1.78 × 10^−10^	7.19 × 10-^−7^
TCR	Co	Cr ^a^	Ni	Pb	CTCR
Pancevo	1.90× 10^−8^	3.10 × 10^−5^	4.44 × 10^−9^	6.11 × 10^−7^	3.17 × 10^−5^
Smederevo	2.05× 10^−8^	5.89 × 10^−5^	7.53 × 10^−9^	1.30 × 10^−6^	6.03 × 10^−5^
Obrenovac	2.55× 10^−8^	3.46 × 10^−5^	5.48 × 10^−9^	6.99 × 10^−7^	3.54 × 10^−5^
Belgrade I	1.81× 10^−8^	2.08 × 10^−5^	3.23 × 10^−9^	4.29 × 10^−6^	2.51 × 10^−5^
Belgrade II	2.24× 10^−8^	2.59 × 10^−5^	3.69 × 10^−9^	8.06 × 10^−7^	2.67 × 10^−5^

CR—carcinogenic risk; CCR—cumulative CR; TCR—total CR; CTCR—cumulative TCR. ^a^ Cr(VI).

**Table 8:** Correlation matrix for metal concentrations.

Element	Co	Cr	Cu	Fe	Mn	Ni	Pb	Sr	Zn
Co	1								
Cr	0.247^.^	1							
Cu	−0.164	0.168	1						
Fe	0.828 **	0.090	−0.183	1					
Mn	0.810 **	0.164	−0.222	0.831 **	1				
Ni	0.349 *	0.954 **	0.171	0.185	0.249	1			
Pb	0.234	0.150	0.796**	−0.147	−0.265	−0.149	1		
Sr	−0.319 *	−0.214	0.097	−0.493 **	−0.539 **	−0.170	0.097	1	
Zn	−0.347 *	0.121	0.841**	−0.435 **	−0.452 **	0.080	0.750 **	0.311	1

* *p* < 0.05; ** *p* < 0.01;

**Table 9:** PCA results (Varimax normalized).

Element	PC1	PC2	PC3
Co	0.856	−0.129	0.198
Cr	0.101	0.035	0.982
Cu	−0.078	0.943	0.160
Fe	0.939	−0.108	−0.001
Mn	0.916	−0.185	0.090
Ni	0.184	0.036	0.968
Pb	−0.057	0.929	−0.199
Sr	−0.635	0.065	−0.104
Zn	−0.359	0.869	0.138
Eigenvalues	3.773	2.348	1.538
Variance %	41.92	26.09	17.08
Cumulative %	41.92	68.01	85.09

## Data Availability

Not applicable.

## Supplementary Materials

The following are available online at https://www.mdpi.com/article/10.3390/ijerph18179412/s1, Table S1: Comparison of analyzed and certified values of BCR-701; Table S2: Background values of PTEs (mg kg^−1^ d.w.) in the studied urban soils (method: Median + 2MAD); Table S3: Classification of Enrichment factor (EF), Contamination factor (Cf), Degree of contamination (Cdeg), Potential ecological risk (E_r_^i^) and potential ecological risk index (RI); Table S4: Description and values of all parameters associated with health risk assessment for PTEs in soils; Table S5: Relative bioavailability factor (RBA), oral reference dose (RfDo), dermal absorption fraction (ABSd), gastrointestinal absorption (GIABS), inhalation reference concentration (RfC), oral slope factor (CSFo), and inhalation unit risk (IUR) values for each PTE; Table S6: Pseudo-total PTE concentrations in the studied urban soils (mg kg^−1^ d.w.); Table S7: Enrichment factor (EF) of PTEs in the studied urban soils; Table S8: Contamination factor (Cf) of PTEs in the studied urban soils; Table S9: Ecological risk index (Ei) of PTEs in the studied urban soils; Table S10: Degree of contamination (Cdeg) and Potential Ecological risk (RI) of PTEs in the studied urban soils, Figure S1: Fractionation profile of Co, Cr and Cu in the studied soils; Figure S2: Fractionation profile of Fe, Mn and Ni in the studied soils; Figure S3: Fractionation profile of Pb, Sr and Zn in the studied soils.
