# Supplementary material for: Chemical Fractionation, Environmental, and Human Health Risk Assessment of Potentially Toxic Elements in Soil of Industrialised Urban Areas in Serbia
Source: Int J Environ Res Public Health. 2021 Sep 6;18(17):9412. doi: 10.3390/ijerph18179412 (PMC8430938; doi:10.3390/ijerph18179412)
Supplement: Supplementary file 1 [file ijerph-18-09412-s001.zip › ijerph-1354712-supplementary.pdf]

Supplementary Information

For

Chemical fractionation, environmental and human health risk assessment of potentially toxic elements in soil of industrialised urban areas in Serbia

Dragana Pavlović[1, *], Marija Pavlović[1], Veljko Perović[1], Zorana Mataruga[1], Dragan Čakmak[1], Miroslava Mitrović[1], Pavle Pavlović[1]

[1]Department of Ecology, Institute for Biological Research 'Siniša Stanković', University of Belgrade, Bulevar despota Stefana 142, 11000 Belgrade, Serbia

E-mail: dragana.pavlovic@ibiss.bg.ac.rs

Tel: +38111 2078358

Fax: +381 11 2761433

*Corresponding author, https://orcid.org/0000-0002-3624-706X

Tables:

Table S1. Comparison of analyzed and certified values of BCR-701.

Table S2. Background values of PTEs (mg kg^{-1} d.w.) in the studied urban soils (method: Median + 2MAD).

Table S3. Classification of Enrichment factor (EF), Contamination factor (Cf), Degree of contamination (Cdeg), Potential ecological risk (E_r^i) and potential ecological risk index (RI).

Table S4. Description and values of all parameters associated with health risk assessment for PTEs in soils.

Table S5. Relative bioavailability factor (RBA), oral reference dose (RfDo), dermal absorption fraction (ABSd), gastrointestinal absorption (GIABS), inhalation reference concentration (RfC), oral slope factor (CSFo), and inhalation unit risk (IUR) values for each PTE.

Table S6. Pseudo-total PTE concentrations in the studied urban soils (mg kg^{-1} d.w.).

Table S7. Enrichment factor (EF) of PTEs in the studied urban soils.

Table S8. Contamination factor (Cf) of PTEs in the studied urban soils.

Table S9. Ecological risk index (Ei) of PTEs in the studied urban soils.

Table S10**.** Degree of contamination (Cdeg) and Potential Ecological risk (RI) of PTEs in the studied urban soils.

Figures:

Figure S1. Fractionation profile of Co, Cr and Cu in the studied soils.

Figure S2. Fractionation profile of Fe, Mn and Ni in the studied soils.

Figure S3. Fractionation profile of Pb, Sr and Zn in the studied soils.

The study area and soil sampling

The study was conducted at five sites exposed to different sources of pollution: Pancevo, Smederevo, Obrenovac, Belgrade and the Arboretum of the Faculty of Forestry (Control site).

Pancevo is located in the southern part of the Autonomous Province of Vojvodina, 20 km from the Serbian capital, Belgrade, between 20°40'00''E and 44°54'00''N, at an average altitude of about 77 m above sea level. Pancevo has a moderate continental climate, characterised by cold winters and long and hot summers. The average annual temperature of Pancevo is 11.3 °C and mean annual precipitation is 643 mm. It covers an area of 148.8 km^2 and has a population of about 76000. Pancevo is known as the centre of the Serbian chemical industry, but also as an environmental hotspot because of the high concentration of air pollutants that makes life unbearable for its inhabitants. The major pollution sources in Pancevo are the Pancevo Oil Refinery, the Pancevo Nitric Fertilizer Factory and the Pancevo Petrochemical Industry. Sampling was carried out in the National Garden (20°39'23''E and 44°51'54''N), the largest and oldest park in Pancevo, located 1.3 km from the town centre. The distance from the industrial zone to the National Garden is approximately 5 km.

Smederevo is located in central Serbia, 46 km from the Serbian capital, Belgrade, between 20°57'00''E and 40°39'00''N, at an average altitude of about 73 m above sea level. Smederevo has a moderate

continental climate, characterised by hot summers with high precipitation in June and November and cold dry winters. The average annual temperature of Smederevo is 11.9 °C and mean annual precipitation is 650 mm. It covers an area of 484 km^2 (urban area: 38.58 km^2) and has a population of about 64000 in the urban area of the town. Smederevo is one of the most densely populated towns in the Republic of Serbia. This high concentration of population, industry and transport, as well as agricultural production, has caused an increased degree of land, air and water degradation and pollution. The main source of pollution in Smederevo is the ironworks, located in the industrial zone 7 km southeast of the city centre. The steel plant produces iron in two blast furnaces and then converts the blast furnace iron into raw steel at a three-furnace converter shop and continuous casting complex. Most pollutants are related to agglomeration and the converter steel plant. Sampling was carried out in the central city zone in the National Heroes Park (20°55'33''E and 44°40'00''N).

Obrenovac is located in the north-west part of Serbia, between 20°12'00''E and 44°39'00''N, at an average altitude of about 76 m above sea level. It is one of 17 municipalities of Belgrade and is located at the confluence of the Kolubara and Sava rivers. Obrenovac has a moderate continental climate, characterised by hot summers with high precipitation in May, June and July and a minimum in October and cold winters. The average annual temperature of Obrenovac is 11.0 °C and mean annual precipitation is 647.2 mm. It covers an area of 40.996 km^2 and has a population of about 72000. The main source of pollution in Obrenovac is the thermoelectric power plant "Nikola Tesla A" (TENT A), situated on the right bank of the Sava River, 42 km upstream from the Serbian capital, Belgrade. TENT A produces the largest amount of fly ash (around 3.6 Mt a year) in Serbia. The sampling site in Obrenovac was in the main City park (20°12'50''E and 44°39'16''N), located 4 km away from the source of pollution (the thermoelectric power plant and the fly ash disposal site).

Belgrade is located in south-eastern Europe, on the Balkan Peninsula, between 20°27'44''E and 44°49'14''N, at an average altitude of about 120 m above sea level. Belgrade has a moderate continental climate, with four seasons characterised by cold winters and hot, humid summers with well distributed rainfall. The average annual temperature of Belgrade is 11.9 °C and mean annual precipitation is 685 mm. It covers an area of 359.96 km^2 and has a population of about 1.2 million in the inner city area. The major pollution sources in Belgrade are heating plants powered by crude oil or natural gas, domestic heating (using coal and crude oil as fuel), gasoline and diesel vehicle exhaust emissions, as well as other vehicle emissions. Traffic has been recognized as the main source of air pollution in the central area of Belgrade. Sampling was carried out at two locations in Belgrade: Hall Pioneer Park (20°28'58''E and 44°48'51''N) in the central zone of the city, one of the most polluted areas of the city, and the Arboretum of the Faculty of Forestry – the control site (20°25'23''E and 44°46'57''N), which is a protected natural area and a valuable archive of domestic and foreign tree species in Belgrade. It is located in an area without a direct source of pollution, 10 km away from the city centre, within a zone of mixed *Quercus frainetto* and *Quercus cerris* forest .

At each sampling site, the soil was sampled at eight sampling points. The sampling sites were chosen based on proximity of industrial plants and heavy traffic.

Table S1. Comparison of analyzed and certified values of BCR-701.

		Cd	Cr	Cu	Ni	Pb	Zn
Step 1	Analyzed	6.7±0.37	2.03±0.12	48.3±1.4	14.9±0.8	2.98±0.22	201.7±6.2
	Certified	7.3±0.4	2.26±0.16	49.3±1.7	15.4±0.9	3.18±0.21	205±6
	% Recovery	91.8	89.8	97.9	96.7	93.7	98.4
Step 2	Analyzed	3.65±0.31	44.9±3.1	126.1±4	27.2±1.5	127.7±3.2	120.4±5.9
	Certified	3.77±0.28	45.7±2.0	124±3	26.6±1.3	126±3	114±5
	% Recovery	96.8	98.24	101.7	102.2	101.3	105.6
Step 3	Analyzed	0.23±0.08	137.8±6.9	60.9±4.2	16.7±1.1	8.7±1.8	48.8±4.3
	Certified	0.27±0.06	143±7	55±4.0	15.3±0.9	9.3±2.0	46±4.0
	% Recovery	85.1	96.4	110.7	109.2	93.5	106.0

Table S2. Background values of PTEs (mg kg^{-1} d.w.) in the studied urban soils (Mrvić et al. 2009; 2011; Knežević 2014).

City	Co	Cr	Cu	Fe	Mn	Ni	Pb	Sr	Zn
Pancevo	6.73	47.51	37.80	50351.84	689.55	49.32	25.11	77.79	96.45
Smederevo	6.97	69.00	58.50	23911.08	620.52	103.00	53.50	137.91	115.00
Obrenovac	8.76	79.00	28.00	41266.52	863.70	131.00	35.12	107.31	74.00
Belgrade	6.74	61.00	30.00	24771.00	489.29	72.00	37.00	223.41	68.00

Table S3. Classification of Enrichment factor (EF), Contamination factor (Cf), Degree of contamination (Cdeg), Potential ecological risk (E_r^i) and potential ecological risk index (RI).

**EF**		**Cf**		**Cdeg**		**Er^i**		**RI**	
EF <2	Minimal enrichment	< 1	low contamination	<8	low degree of contamination	<40	Low potential ecological risk	<150	Low ecological risk
2 ≤ EF < 5	Moderate enrichment	1 - 3	moderate contamination	8-16	moderate degree of contamination	40 ≤ Er^i < 80	Moderate potential ecological risk	150-300	Moderate ecological risk
5 ≤ EF < 20	Significant enrichment	3 - 6	considerable contamination	16-32	considerable degree of contamination	80 ≤ Er^i < 160	Considerable potential ecological risk	300-600	Considerable ecological risk
20 ≤ Ef < 40	Very high enrichment	> 6	very high contamination	>32	very high degree of contamination	160 ≤ Er^i < 320	High potential ecological risk	>600	Very high ecological risk
≥ 40	Extremely high enrichment					≥ 320	Very high potential ecological risk		
Source	Chen et al., (2019)				Hakanson, (1980)				

Table S4. Description and values of all parameters associated with health risk assessment for PTEs in soils.

Symbol	Parameters (units)	Values	References
C	PTE concentration (mg/kg)		Site specific
IRS_{res-c}	Resident Soil Ingestion Rate - Child (mg/day)	200	USEPA, 2020a
IRS_{res-a}	Resident Soil Ingestion Rate - Adult (mg/day)	100	USEPA, 2020a
RBA	relative bioavailability factor	Arsenic=0.6<br>All Others=1	USEPA, 2020a
EF_{res}	Resident Exposure Frequency Adult, Child (days/year)	350	USEPA, 2020a
ED_{res-c}	Resident Exposure Duration - child (years)	6	USEPA, 2020a
ED_{res-a}	Resident Exposure Duration - adult (years)	20	ED_{res} (26 years)-<br>ED_{res-c} (6 years)<br>USEPA, 2020a
BW_{res-c}	Resident Body Weight - child (kg)	15	USEPA, 2020a
BW_{res-a}	Resident Body Weight - adult (kg)	80	USEPA, 2020a
AT_{res-c}	Averaging time - resident child (days)	365 x ED_{res-c}=2190	USEPA, 2020a
AT_{res-a}	Averaging time - resident adult (days)	365 x ED_{res} =7300	USEPA, 2020a
RfD_o	Chronic Oral Reference Dose (mg/kg-day)	Contaminant specific	Table S5
SA_{res-c}	Resident surface area soil - child (cm^2/day)	2373	USEPA, 2020a
SA_{res-a}	Resident surface area soil - adult (cm^2/day)	6032	USEPA, 2020a
AFa	Skin adherence factor - adult (mg/cm^2)	0,07	USEPA, 2020a
AFc	Skin adherence factor - child (mg/cm^2)	0,2	USEPA, 2020a
ABSd	Fraction of contaminant absorbed dermally from soil (unitless)	Contaminant specific	Table S5
GIABS	Fraction of contaminant absorbed in gastrointestinal tract (unitless) Note: if GIABS is >50% then it is set to 100% for calculation of dermal toxicity values	Contaminant specific<br>Inorganic default=1.0<br>VOC default=1.0<br>SVOC default=1.0	Table S5
RfC	Chronic Inhalation Reference Concentration (mg/m^3)	Contaminant specific	Table S5
PEF	Particulate Emission Factor - Minneapolis (m^3/kg)	1.36 x 10^9(region-specific)	USEPA, 2020a
$IFS_{res-adj}$	Resident Soil Ingestion Rate - age-adjusted (mg/kg)	Calculated using the age adjusted intake factors equation 36750	USEPA, 2020a
$DFS_{res-adj}$	Resident soil dermal contact factor- age-adjusted (mg/kg)	Calculated using the age adjusted intake factors equation 103390	USEPA, 2020a
CSFo	Oral Slope Factor (mg/kg-day)$^{-1}$		Table S5
IUR	Inhalation Unit Risk (µg/m^3)$^{-1}$	Contaminant specific	Table S5
LT	Life time (years)	76	Site specific
AT	Averaging time (days)	365*LT=27740 (Carcinogenic)	Site specific

Table S5. Relative bioavailability factor (RBA), oral reference dose (RfDo), dermal absorption fraction (ABSd), gastrointestinal absorption (GIABS), inhalation reference concentration (RfC), oral slope factor (CSFo), and inhalation unit risk (IUR) values for each PTE.

Metal	RBA	RfDo	ABSd	GIABS	RfC	CSFo	IUR
**Co**	1	0.0003	0.001	1	0.000006	/	0.009
**Cr (VI)**	1	0.003	0.001	0.025	0.0001	0.5	0.0840
**Cu**	1	0.04	0.001	1	0.0024[b,c]	/	/
**Fe**	1	0.7	0.001	1	/	/	/
**Mn**	1	0.024	0.001	0.04	0.000050	/	/
**Ni**	1	0.02	0.001	0.04	0.000090	/	0.0003
**Pb**	1	0.0014[a]	0.001	1	0.0015[b,c]	0.0085[b,c]	0.000012[b,c]
**Sr**	1	0.6	0.001	1	/	/	/
**Zn**	1	0.3	0.001	1	/ 0.035[b,c]	/	/
**Source**				USEPA (2020b)			

[a] Jia et al., (2018); [b] USDOE, (2011); [c] Čakmak et al., (2020)

Table S6. Pseudo-total PTEs concentrations in the studied urban soils (mg kg^{-1} d.w.).

Sampling point	Co	Cr	Cu	Fe	Mn	Ni	Pb	Sr	Zn
Pancevo 1	8.96	41.40	30.18	33209.92	595.58	56.90	45.21	31.61	39.45
Pancevo 2	8.85	39.55	34.48	33390.68	572.30	51.10	55.86	100.59	50.30
Pancevo 3	8.12	36.17	29.41	31093.92	551.87	60.48	40.24	24.08	51.52
Pancevo 4	8.78	45.52	32.80	34410.49	602.50	76.92	47.39	33.60	45.24
Pancevo 5	9.17	48.37	32.81	33116.43	585.81	69.50	47.66	27.93	38.17
Pancevo 6	8.72	37.87	29.92	31836.19	544.47	57.59	50.09	48.24	44.23
Pancevo 7	8.23	41.87	28.47	32297.32	499.21	63.80	43.22	45.65	56.62
Pancevo 8	9.25	36.97	30.87	33389.75	611.98	54.49	42.81	27.97	38.92
Smederevo 1	9.87	88.61	48.56	30462.99	493.59	101.04	82.57	67.43	107.23
Smederevo 2	10.29	71.31	48.35	30844.47	554.40	95.92	57.27	66.48	168.72
Smederevo 3	9.16	87.18	45.46	28940.54	473.33	125.60	200.18	73.52	92.58
Smederevo 4	9.80	98.82	48.64	30789.74	597.23	134.34	93.60	57.21	106.12
Smederevo 5	12.09	79.87	39.03	40037.83	535.74	113.01	84.12	89.46	86.47
Smederevo 6	6.72	72.05	30.58	18117.88	342.22	88.78	58.74	84.11	112.08
Smederevo 7	8.43	49.46	43.63	26358.68	514.91	57.41	70.10	62.18	117.68
Smederevo 8	9.31	75.49	84.07	29931.15	556.63	116.33	146.32	60.08	127.66
Obrenovac 1	14.69	54.00	35.01	41141.78	912.85	85.38	53.78	31.45	43.76
Obrenovac 2	13.12	56.72	42.95	36855.54	664.16	92.20	61.44	50.16	70.94
Obrenovac 3	10.29	38.37	30.80	32119.40	546.76	66.05	47.83	131.18	38.39
Obrenovac 4	13.11	52.37	36.62	38475.96	706.12	88.65	69.21	47.99	50.91
Obrenovac 5	8.13	28.20	23.81	25939.71	453.31	51.32	47.74	155.61	34.82
Obrenovac 6	11.53	50.59	31.78	34588.45	570.63	84.27	52.41	103.58	56.97
Obrenovac 7	11.75	45.73	33.54	34641.16	640.29	75.70	53.26	84.58	48.30
Obrenovac 8	11.40	39.85	33.82	33367.29	628.24	62.01	40.44	72.06	42.30
Belgrade I 1	7.99	25.53	38.36	25315.29	407.21	43.15	122.74	173.51	140.41
Belgrade I 2	8.89	25.71	33.84	28728.79	476.85	47.81	56.71	190.49	60.64
Belgrade I 3	8.33	26.75	36.68	28325.58	477.74	42.68	52.69	62.20	91.58
Belgrade I 4	6.48	28.01	53.47	23315.14	391.74	43.90	269.40	135.20	139.51
Belgrade I 5	7.77	23.28	107.36	29617.86	423.03	38.37	1750.38	80.54	302.36
Belgrade I 6	9.07	30.28	38.33	29738.63	546.83	38.55	104.45	74.06	94.83
Belgrade I 7	8.93	35.89	55.79	26735.91	418.21	61.07	193.17	163.97	199.64
Belgrade I 8	9.25	24.41	34.92	29574.29	474.49	41.25	66.69	97.97	53.70
Belgrade II 1	10.52	34.21	32.90	34671.38	513.88	49.80	72.15	56.61	46.32
Belgrade II 2	11.35	36.37	29.42	34566.13	627.63	52.81	49.05	28.45	39.48
Belgrade II 3	10.48	32.33	29.98	36066.42	627.79	43.69	50.53	28.86	45.83
Belgrade II 4	10.34	39.85	36.62	29409.41	477.58	61.30	94.13	30.32	47.94
Belgrade II 5	10.33	36.38	35.29	33402.31	568.50	67.75	65.30	39.05	68.23
Belgrade II 6	8.77	22.78	33.86	30486.61	441.45	34.55	46.72	92.49	37.80
Belgrade II 7	10.13	39.73	33.37	33130.86	546.63	52.67	60.86	39.71	54.63
Belgrade II 8	10.62	31.75	44.31	34445.66	581.93	45.17	52.62	36.02	48.22

Table S7. Enrichment factor (EF) of PTEs in the studied urban soils.

Sampling point	Co	Cr	Cu	Mn	Ni	Pb	Sr	Zn
Pancevo 1	2.018	1.321	1.211	1.310	1.749	2.729	0.616	0.620
Pancevo 2	1.984	1.255	1.376	1.252	1.562	3.354	1.950	0.786
Pancevo 3	1.954	1.233	1.260	1.296	1.986	2.594	0.501	0.865
Pancevo 4	1.910	1.402	1.270	1.279	2.282	2.761	0.632	0.686
Pancevo 5	2.072	1.548	1.320	1.292	2.143	2.886	0.546	0.602
Pancevo 6	2.050	1.261	1.252	1.249	1.847	3.155	0.981	0.725
Pancevo 7	1.907	1.374	1.174	1.129	2.017	2.683	0.915	0.915
Pancevo 8	2.072	1.173	1.232	1.338	1.666	2.570	0.542	0.608
Smederevo 1	1.112	1.008	0.651	0.553	0.770	1.211	0.384	0.732
Smederevo 2	1.145	0.801	0.641	0.613	0.722	0.830	0.374	1.137
Smederevo 3	1.086	1.044	0.642	0.558	1.007	3.091	0.440	0.665
Smederevo 4	1.091	1.112	0.646	0.662	1.013	1.359	0.322	0.717
Smederevo 5	1.036	0.691	0.398	0.456	0.655	0.939	0.387	0.449
Smederevo 6	1.272	1.378	0.690	0.644	1.138	1.449	0.805	1.286
Smederevo 7	1.098	0.650	0.677	0.666	0.506	1.189	0.409	0.928
Smederevo 8	1.066	0.874	1.148	0.634	0.902	2.185	0.348	0.887
Obrenovac 1	1.197	0.686	1.254	1.060	0.654	1.536	0.294	0.593
Obrenovac 2	1.193	0.804	1.717	0.861	0.788	1.959	0.523	1.073
Obrenovac 3	1.074	0.624	1.413	0.813	0.648	1.750	1.571	0.667
Obrenovac 4	1.142	0.711	1.403	0.877	0.726	2.113	0.480	0.738
Obrenovac 5	1.051	0.568	1.353	0.835	0.623	2.162	2.307	0.749
Obrenovac 6	1.117	0.764	1.354	0.788	0.767	1.780	1.152	0.919
Obrenovac 7	1.137	0.690	1.427	0.883	0.688	1.806	0.939	0.778
Obrenovac 8	1.145	0.624	1.494	0.900	0.585	1.424	0.830	0.707
Beograd I 1	1.160	0.409	1.251	0.814	0.586	3.246	0.760	2.020
Beograd I 2	1.137	0.363	0.973	0.840	0.572	1.322	0.735	0.769
Beograd I 3	1.080	0.383	1.069	0.854	0.518	1.245	0.243	1.178
Beograd I 4	1.021	0.488	1.894	0.851	0.648	7.736	0.643	2.180
Beograd I 5	0.964	0.319	2.993	0.723	0.446	39.566	0.302	3.719
Beograd I 6	1.120	0.413	1.064	0.931	0.446	2.351	0.276	1.162
Beograd I 7	1.228	0.545	1.723	0.792	0.786	4.837	0.680	2.720
Beograd I 8	1.149	0.335	0.975	0.812	0.480	1.510	0.367	0.661
Beograd II 1	1.115	0.401	0.783	0.750	0.494	1.393	0.181	0.487
Beograd II 2	1.207	0.427	0.703	0.919	0.526	0.950	0.091	0.416
Beograd II 3	1.068	0.364	0.686	0.881	0.417	0.938	0.089	0.463
Beograd II 4	1.292	0.550	1.028	0.822	0.717	2.143	0.114	0.594
Beograd II 5	1.137	0.442	0.872	0.862	0.698	1.309	0.130	0.744
Beograd II 6	1.057	0.303	0.917	0.733	0.390	1.026	0.336	0.452
Beograd II 7	1.124	0.487	0.832	0.835	0.547	1.230	0.133	0.601
Beograd II 8	1.134	0.374	1.062	0.855	0.451	1.023	0.116	0.510

Table S8. Contamination factor (Cf) of PTEs in the studied urban soils.

Sampling point	Co	Cr	Cu	Fe	Mn	Ni	Pb	Sr	Zn
Pancevo 1	1.331	0.871	0.237	0.660	0.864	1.154	1.800	0.406	0.207
Pancevo 2	1.315	0.833	0.234	0.663	0.830	1.036	2.224	1.293	0.260
Pancevo 3	1.207	0.761	0.215	0.618	0.800	1.226	1.602	0.310	0.276
Pancevo 4	1.305	0.958	0.232	0.683	0.874	1.560	1.887	0.432	0.216
Pancevo 5	1.363	1.018	0.243	0.658	0.850	1.409	1.898	0.359	0.239
Pancevo 6	1.296	0.797	0.231	0.632	0.790	1.168	1.995	0.620	0.211
Pancevo 7	1.223	0.881	0.218	0.641	0.724	1.294	1.721	0.587	0.506
Pancevo 8	1.374	0.778	0.245	0.663	0.888	1.105	1.704	0.360	0.407
Smederevo 1	1.417	1.284	0.169	1.274	0.704	0.981	1.543	0.867	0.498
Smederevo 2	1.477	1.033	0.176	1.290	0.791	0.931	1.070	0.855	0.565
Smederevo 3	1.314	1.263	0.157	1.210	0.675	1.219	3.742	0.945	0.456
Smederevo 4	1.405	1.432	0.167	1.288	0.852	1.304	1.750	0.735	0.412
Smederevo 5	1.734	1.158	0.207	1.674	0.764	1.097	1.572	1.150	0.283
Smederevo 6	0.964	1.044	0.115	0.758	0.488	0.862	1.098	1.081	0.431
Smederevo 7	1.210	0.717	0.144	1.102	0.735	0.557	1.310	0.799	0.309
Smederevo 8	1.335	1.094	0.159	1.252	0.794	1.129	2.735	0.772	0.324
Obrenovac 1	1.677	0.684	0.525	0.997	1.057	0.652	1.531	0.404	0.219
Obrenovac 2	1.498	0.718	0.469	0.893	0.769	0.704	1.749	0.645	0.299
Obrenovac 3	1.175	0.486	0.368	0.778	0.633	0.504	1.362	1.686	0.161
Obrenovac 4	1.496	0.663	0.468	0.932	0.818	0.677	1.970	0.617	0.289
Obrenovac 5	0.928	0.357	0.290	0.629	0.525	0.392	1.359	2.000	0.261
Obrenovac 6	1.316	0.640	0.412	0.838	0.661	0.643	1.492	1.332	0.228
Obrenovac 7	1.342	0.579	0.420	0.839	0.741	0.578	1.516	1.087	0.146
Obrenovac 8	1.301	0.504	0.407	0.809	0.727	0.473	1.151	0.926	0.147
Beograd I 1	1.185	0.418	0.266	1.022	0.832	0.599	3.317	2.230	0.153
Beograd I 2	1.319	0.421	0.296	1.160	0.975	0.664	1.533	2.449	0.160
Beograd I 3	1.235	0.438	0.277	1.143	0.976	0.593	1.424	0.800	0.133
Beograd I 4	0.961	0.459	0.216	0.941	0.801	0.610	7.281	1.738	0.173
Beograd I 5	1.153	0.382	0.259	1.196	0.865	0.533	47.308	1.035	0.205
Beograd I 6	1.345	0.496	0.302	1.201	1.118	0.535	2.823	0.952	0.139
Beograd I 7	1.325	0.588	0.298	1.079	0.855	0.848	5.221	2.108	0.195
Beograd I 8	1.372	0.400	0.308	1.194	0.970	0.573	1.803	1.259	0.208
Beograd II 1	1.561	0.561	0.351	1.400	1.050	0.692	1.950	0.728	0.185
Beograd II 2	1.684	0.596	0.378	1.395	1.283	0.733	1.326	0.366	0.228
Beograd II 3	1.555	0.530	0.349	1.456	1.283	0.607	1.366	0.371	0.208
Beograd II 4	1.533	0.653	0.345	1.187	0.976	0.851	2.544	0.390	0.130
Beograd II 5	1.533	0.596	0.344	1.348	1.162	0.941	1.765	0.502	0.227
Beograd II 6	1.301	0.373	0.292	1.231	0.902	0.480	1.263	1.189	0.181
Beograd II 7	1.503	0.651	0.338	1.337	1.117	0.731	1.645	0.511	0.000
Beograd II 8	1.576	0.521	0.354	1.391	1.189	0.627	1.422	0.463	0.000

Table S9. Ecological risk index (Ei) of PTEs in the studied urban soils.

Sampling point	Cr	Cu	Ni	Pb	Zn
Pancevo 1	1.743	1.185	5.769	9.001	0.207
Pancevo 2	1.665	1.171	5.180	11.122	0.260
Pancevo 3	1.523	1.074	6.132	8.011	0.276
Pancevo 4	1.916	1.162	7.799	9.435	0.216
Pancevo 5	2.036	1.213	7.046	9.489	0.239
Pancevo 6	1.594	1.154	5.839	9.973	0.211
Pancevo 7	1.763	1.089	6.468	8.606	0.506
Pancevo 8	1.556	1.223	5.524	8.522	0.407
Smederevo 1	2.568	0.844	4.905	7.717	0.498
Smederevo 2	2.067	0.880	4.656	5.352	0.565
Smederevo 3	2.527	0.783	6.097	18.708	0.456
Smederevo 4	2.864	0.837	6.521	8.748	0.412
Smederevo 5	2.315	1.033	5.486	7.862	0.283
Smederevo 6	2.088	0.574	4.310	5.489	0.431
Smederevo 7	1.434	0.721	2.787	6.552	0.309
Smederevo 8	2.188	0.795	5.647	13.675	0.324
Obrenovac 1	1.367	2.623	3.259	7.656	0.219
Obrenovac 2	1.436	2.343	3.519	8.746	0.299
Obrenovac 3	0.971	1.838	2.521	6.809	0.161
Obrenovac 4	1.326	2.340	3.383	9.852	0.289
Obrenovac 5	0.714	1.452	1.959	6.795	0.261
Obrenovac 6	1.281	2.059	3.216	7.460	0.228
Obrenovac 7	1.158	2.099	2.889	7.582	0.146
Obrenovac 8	1.009	2.036	2.367	5.757	0.147
Beograd I 1	0.837	1.332	2.996	16.587	0.153
Beograd I 2	0.843	1.481	3.320	7.664	0.160
Beograd I 3	0.877	1.387	2.964	7.120	0.133
Beograd I 4	0.918	1.080	3.049	36.406	0.173
Beograd I 5	0.763	1.295	2.665	236.538	0.205
Beograd I 6	0.993	1.511	2.677	14.115	0.139
Beograd I 7	1.177	1.489	4.241	26.104	0.195
Beograd I 8	0.800	1.541	2.865	9.013	0.208
Beograd II 1	1.122	1.754	3.458	9.750	0.185
Beograd II 2	1.192	1.892	3.667	6.628	0.228
Beograd II 3	1.060	1.746	3.034	6.829	0.208
Beograd II 4	1.306	1.723	4.257	12.720	0.130
Beograd II 5	1.193	1.722	4.705	8.825	0.227
Beograd II 6	0.747	1.461	2.399	6.313	0.181
Beograd II 7	1.303	1.689	3.657	8.224	0.000
Beograd II 8	1.041	1.771	3.137	7.111	0.000

Table S10. Degree of contamination (Cdeg) and Potential Ecological risk (RI) of PTEs in the studied urban soils.

Sampling point	Cdeg	RI
Pancevo 1	7.530	17.904
Pancevo 2	8.689	19.399
Pancevo 3	7.015	17.016
Pancevo 4	8.148	20.529
Pancevo 5	8.036	20.024
Pancevo 6	7.740	18.772
Pancevo 7	7.796	18.432
Pancevo 8	7.524	17.234
Smederevo 1	8.737	16.532
Smederevo 2	8.188	13.520
Smederevo 3	10.983	28.571
Smederevo 4	9.346	19.382
Smederevo 5	9.639	16.978
Smederevo 6	6.841	12.893
Smederevo 7	6.883	11.802
Smederevo 8	9.595	22.629
Obrenovac 1	7.746	15.124
Obrenovac 2	7.743	16.343
Obrenovac 3	7.153	12.300
Obrenovac 4	7.930	17.191
Obrenovac 5	6.742	11.182
Obrenovac 6	7.562	14.244
Obrenovac 7	7.248	13.873
Obrenovac 8	6.447	11.315
Beograd I 1	10.024	21.904
Beograd I 2	8.976	13.468
Beograd I 3	7.020	12.481
Beograd I 4	13.180	41.625
Beograd I 5	52.935	241.467
Beograd I 6	8.911	19.435
Beograd I 7	12.518	33.206
Beograd I 8	8.087	14.427
Beograd II 1	8.477	16.269
Beograd II 2	7.989	13.607
Beograd II 3	7.724	12.877
Beograd II 4	8.610	20.135
Beograd II 5	8.419	16.671
Beograd II 6	7.212	11.102
Beograd II 7	7.834	14.872
Beograd II 8	7.543	13.060

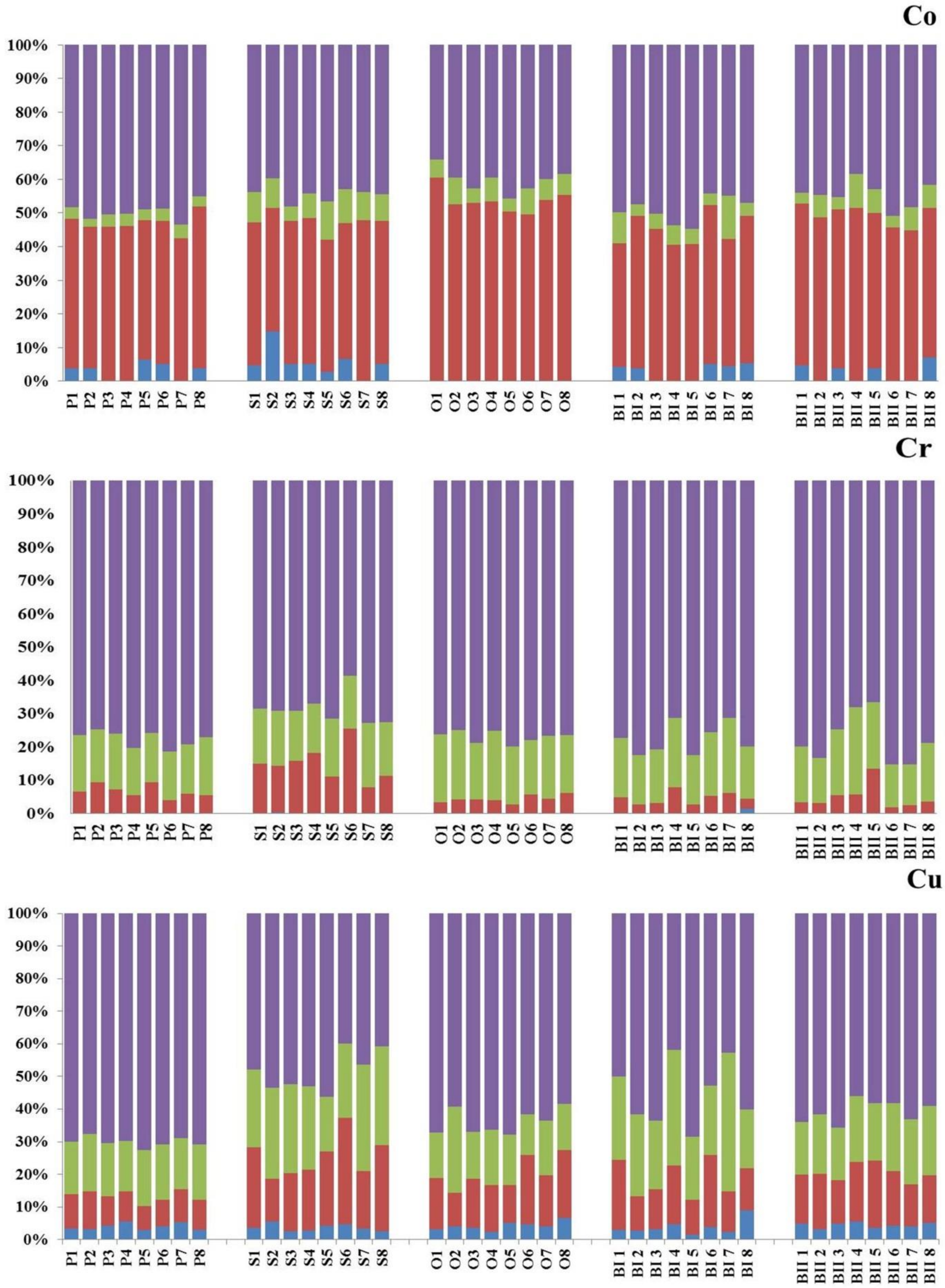

Figure S1. Fractionation profile of Co, Cr and Cu in the studied soils.

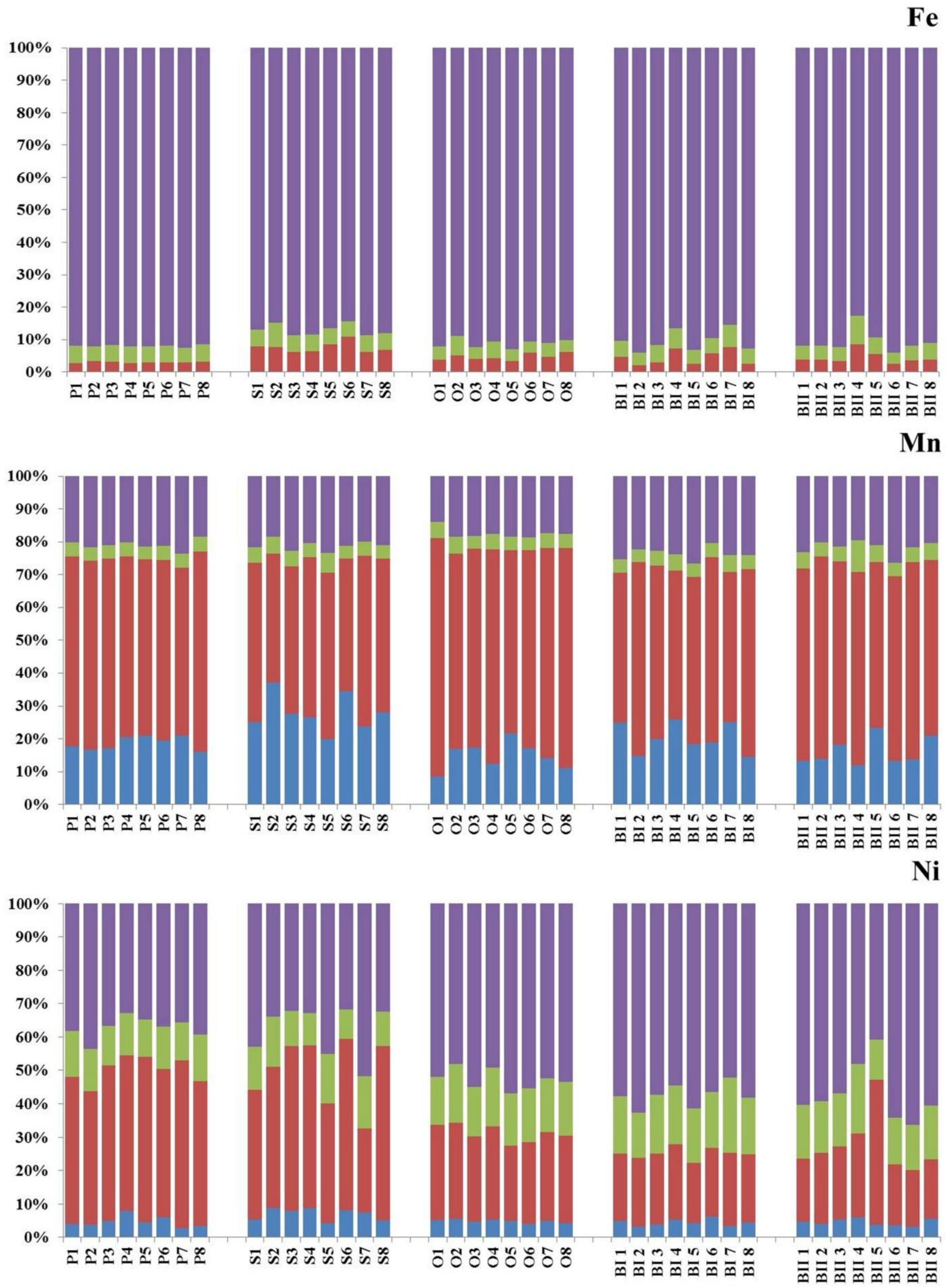

Figure S2. Fractionation profile of Fe, Mn and Ni in the studied soils.

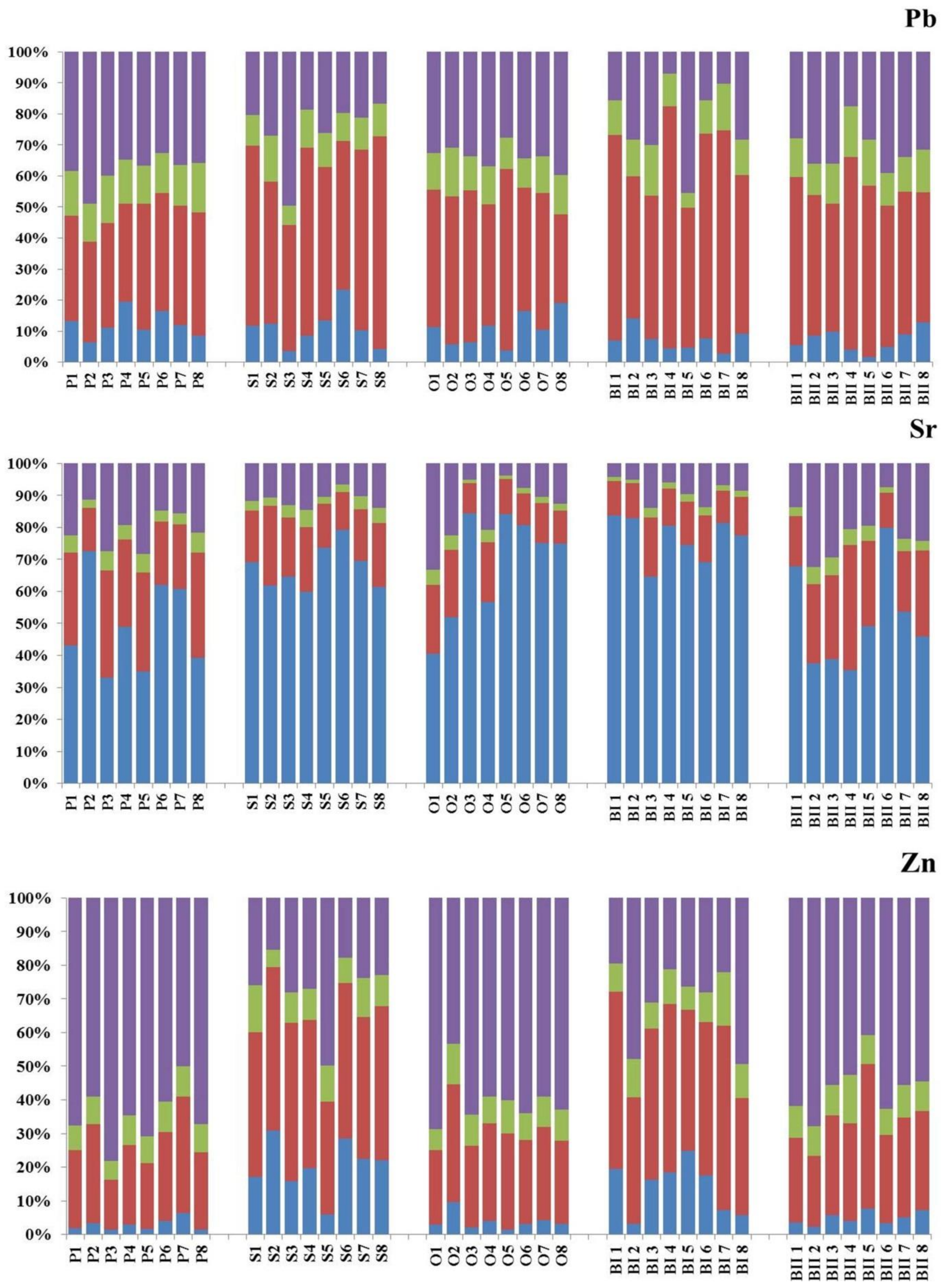

Figure S3. Fractionation profile of Pb, Sr and Zn in the studied soils.

References:

1. Chen, R., Chen, H., Song, L., Yao, Z., Meng, F., Yanguo Teng, Y. (2019). Characterization and source apportionment of heavy metals in the sediments of Lake Tai (China) and its surrounding soils. *Science of the Total Environment, 694*, 133819. https://doi.org/10.1016/j.scitotenv.2019.133819
2. Čakmak, D., Perović, V., Kresović, M., Pavlović, D., Pavlović, M., Mitrović, M., & Pavlović, P. (2020). Sources and health risk assessment of potentially toxic elements in dust at children's playgrounds with artificial surfaces: A case study in Belgrade. *Archives of Environmental Contamination and Toxicology*, *78*, 190-205. https://doi.org/10.1007/s00244-019-00702-0
3. Hakanson, L. (1980). An ecological risk index for aquatic pollution control. A sedimentological approach. *Water Research*, *14*, 975-1001. https://doi.org/10.1016/0043-1354(80)90143-8
4. Imperato, M., Adamo, P., Naimo, D., Arienzo, M., Stanzione, D., Violante, P. (2003). Spatial distribution of heavy metals in urban soils of Naples city (Italy). *Environmental Pollution, 124*, 247-256. https://doi.org/10.1016/S0269-7491(02)00478-5
5. Jia, Z., Li, S., & Wang, L. (2018). Assessment of soil heavy metals for eco-environment and human health in a rapidly urbanization area of the upper Yangtze Basin. *Scientific Reports, 8*, 3256. https://doi.org/10.1038/s41598-018-21569-6
6. Knežević, M. (2014). Determination of the state, degree of pollution and chemical degradation of soil in industrial zones in Pančevo and Šabac, as well as fire sites on Mt. Tara (Project Contract No. 401-00-00051/2014-02). Ministry of agriculture and environmental protection. (in Serbian).
7. Mrvić, V., Zdravković, M., Sikirić, B., Čakmak, D., Kostić-Kravljanac, Lj. (2009). Harmful and hazardous elements in soil. In: V. Mrvić, G. Antonović, L. Martinović (Ed.) *The fertility and content of hazardous and harmful substances in the soils of Central Serbia* (1st ed., pp.75-144). Institute of Soil Science, Belgrade. (in Serbian).
8. Mrvić, V., Kostić-Kravljanac, Lj., Čakmak, D., Sikirić, B., Brebanović, B., Perović, V., & Nikoloski, M. (2011). Pedogeochemical mapping and background limit of trace elements in soils of Branicevo Province (Serbia). *Journal of Geochemical Exploration, 109*, 18-25. https://doi.org/10.1016/j.gexplo.2010.09.005
9. USDOE (2011). The risk assessment information system (RAIS). U.S. Department of Energy's Oak Ridge Operations Office (ORO).
10. USEPA (2020). Regional screening levels (RSLs) - User's Guide. Accessed date: 2 July 2020. https://www.epa.gov/risk/regional-screening-levels-rsls-users-guide.
